# Myelin sheaths in the central nervous system can withstand damage and dynamically remodel

**DOI:** 10.1126/science.adr4661

**Published:** 2026-02-12

**Authors:** Donia Arafa, Julia van de Korput, Philipp N. Braaker, Kieran P. Higgins, Niels R. C. Meijns, Katy L.H. Marshall-Phelps, Julia Meng, Daniel Soong, Eleonora Scalia, Kyle Lathem, Marcus Keatinge, Claire Richmond, Anna Klingseisen, Marja Main, Sarah A. Neely, David W. Hampton, Greg J. Duncan, Geert J Schenk, Marie Louise Groot, Siddharthan Chandran, Ben Emery, Antonio Luchicchi, Maarten H. P. Kole, Anna C. Williams, David A. Lyons

**Affiliations:** 1 Institute for Neuroscience and Cardiovascular Research, University of Edinburgh, Edinburgh EH16 4SB, United Kingdom; 2 MS Society Edinburgh Centre for MS Research, University of Edinburgh, Edinburgh EH16 4SB, United Kingdom; 4 Centre for Regenerative Medicine, Institute of Regeneration and Repair, University of Edinburgh, Edinburgh EH16 4UU, United Kingdom; 6 Department of Axonal Signalling, Netherlands Institute for Neurosciences (NIN), Royal Netherlands Academy for Arts and Sciences (KNAW), 1105 BA Amsterdam, The Netherlands; 7 Cell Biology, Neurobiology and Biophysics, Department of Biology, Faculty of Science, Utrecht University, 3584 CS Utrecht, The Netherlands; 8 Department of Anatomy and Neurosciences, Amsterdam University Medical Center, VU Medical Center, VU University; Amsterdam Neuroscience, Amsterdam University Medical Center; Amsterdam, MS Center Amsterdam, 1081 HZ Amsterdam, The Netherlands; 9 Zebrafish Imaging and Screening Facility, University of Edinburgh, Edinburgh EH16 4SB, United Kingdom; 10 UK Dementia Research Institute at University of Edinburgh, Edinburgh EH16 4SBM, United Kingdom; 11 Euan MacDonald Centre for Motor Neuron Disease Research, Edinburgh EH16 4SB, United Kingdom; 12 Department of Neurology, Jungers Center for Neurosciences Research, Oregon Health & Science University, Portland, Oregon OR 97239, USA; 14 Faculty of Science, Department of Physics, Vrije Universiteit Amsterdam, 1081 HZ Amsterdam, The Netherlands

## Abstract

Myelin damage is a hallmark of several neurological disorders; however, how myelin damage occurs remains to be fully understood. In this study, we find that early damage in zebrafish and rodent demyelination models is characterized by myelin swelling. We show, through live imaging, that myelin swelling does not always lead to myelin loss, and that swellings can sometimes resolve, allowing sheaths to remodel. Increased neuronal activity during early demyelination exacerbates myelin damage, whereas reducing neuronal activity mitigates myelin swelling in both zebrafish and mice. In human multiple sclerosis tissue, myelin swelling is also dynamic, and prominent around active lesions. Our data indicate that myelin swelling is a conserved feature of demyelination and that damage to myelin sheaths can resolve, opening opportunities for targeting human disease.

## Introduction

Myelin, essential for nervous system function, is disrupted and/or lost across a range of human diseases, from genetic disorders of early life through adult neurodegenerative conditions such as multiple sclerosis (MS) (1–3). In certain genetically inherited leukodystrophies, central nervous system (CNS) myelin can exhibit signs of severe damage often manifesting as swelling of and around myelin (4) and myelin swelling is also evident across numerous animal models and conditions where myelin is lost (5–11). This suggests the possibility that the initial process of demyelination may include a period of damage that is characterised by myelin swelling that prefigures myelin loss. In contexts when myelin is lost, a regenerative response called remyelination can often take place, where *de novo* myelin sheaths are made by newly-generated oligodendrocytes or, occasionally, oligodendrocytes that survive myelin loss (12–16). However, what is not yet clear is whether damaged myelin can recover rather than being lost; if myelin does have the potential to withstand damage and remodel, it might provide additional opportunities to treat human conditions in which it is disrupted.

Motivated by the prominent appearance of severe myelin swelling in disease, and by the remarkable adaptability and potential for remodelling of myelin in the healthy nervous system (17–19), we asked whether myelin can withstand damage and recover, pre-empting its loss.

## Results

### Myelin swelling occurs early in a variety of demyelination models

To study early stages of myelin damage that might prefigure myelin loss, we assessed pathology across models used to induce demyelination. We first examined a genetic mouse model of CNS demyelination caused by inducible knockout of the essential transcription factor *Myrf* from mature oligodendrocytes (Myrf^ΔiPlp1,^ see Methods) (20). By electron microscopy, we observed swelling of myelin sheaths in the optic nerve during early stages of demyelination from 8 weeks post-treatment with tamoxifen (Fig. S1A-C’). At 10 weeks post-tamoxifen, Myrf^fl/fl^ control animals had an average of only 0.65 swellings per 10,000 µm^2^ (N = 4, average area analysed per animal =23,245 µm^2^), whereas Myrf^ΔiPlp1^ mutants had 20.1 such swellings per 10,000 µm^2^ (N = 4, average area analysed per animal =28,372 µm^2^).

We next examined myelin pathology by electron microscopy in two genetic zebrafish models of demyelination, one where the bacterial enzyme nitroreductase (NTR) is expressed in oligodendrocytes and activated by the prodrug metronidazole (mtz) and the other in which the rat TRPV1 channel is expressed in oligodendrocytes and activated by capsaicin (csn) (15, 21) (Methods). Similar to observations in the Myrf^ΔiPlp1^ mice, we found that in both zebrafish models, myelin damage was characterized by myelin swelling before extensive myelin loss (Fig. S1D, E). To investigate myelin damage in an inflammatory mouse model of CNS damage, we assessed relapsing-remitting experimental autoimmune encephalomyelitis (EAE). By 29 days post-induction, we observed no myelin loss but widespread myelin alterations in the dorsal spinal cord including the appearance of myelin swelling (Fig. S1F) (22). Given the prevalence of myelin swelling prior to myelin loss across models, we next investigated the dynamics of myelin damage using live imaging approaches and tested whether myelin sheaths had the capacity to withstand damage and remodel.

### Remodelling of myelin sheaths following damage

**Fig. 1:**
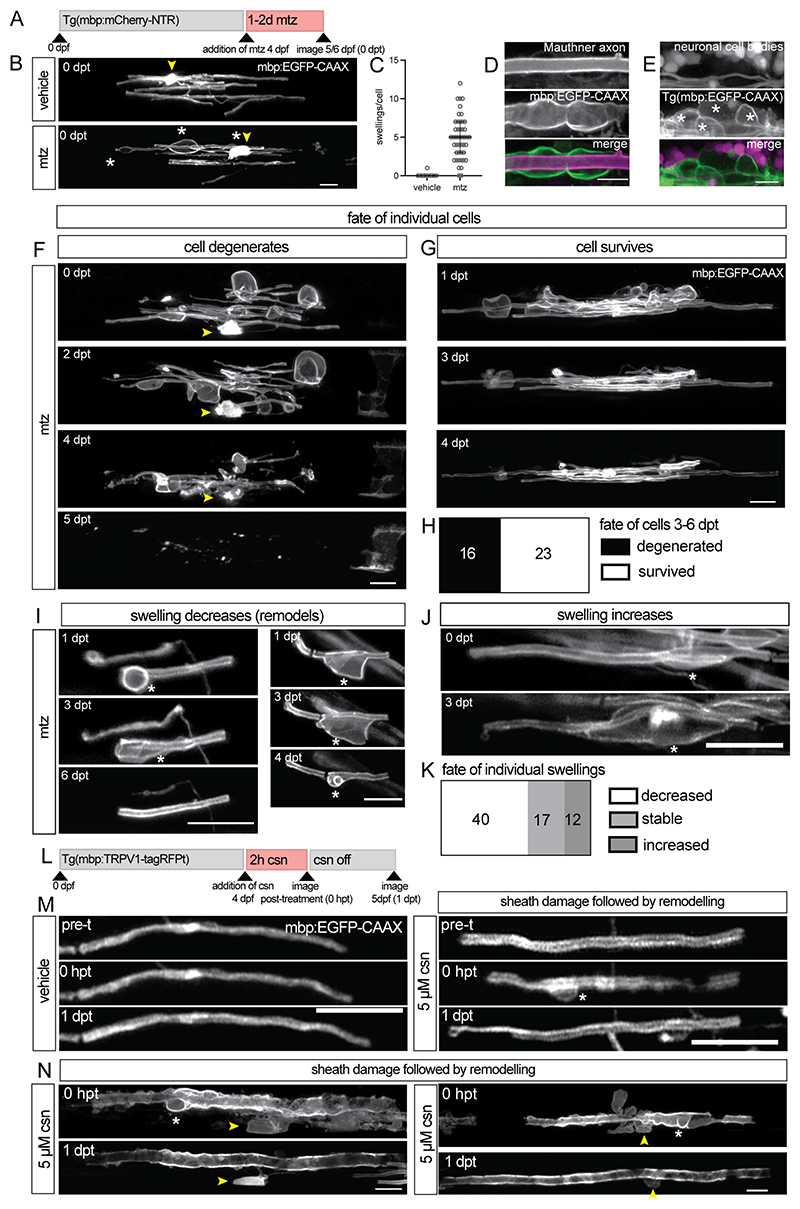
Myelin sheaths can remodel following damage in zebrafish demyelination models **(A)** Schematic of experimental timings for imaging myelin swellings in individual oligodendrocytes in the zebrafish Tg(mbp:mCherry-NTR) model. **(B)** Confocal images of single oligodendrocytes in the spinal cord from animals treated for 1 day with vehicle or 5 mM mtz. Yellow arrows indicate cell body location. Asterisks indicate myelin swellings. Scale bar = 10 µm. **(C)** Quantification of swellings/cell in dorsal and ventral spinal cord oligodendrocytes following 1-2 days treatment with vehicle/5 mM mtz. Error bars are median ± interquartile range (46 cells from 38 mtz-treated animals, 16 cells from 14 vehicle-treated animals). **(D)** Confocal images of myelin (Tg(mbp:EGFP-CAAX)) and the Mauthner axon (Tg(Mauthner:Gal4;UAS:mem-mScarlet)) immediately after 2 days of treatment with 5 mM mtz. Scale bar = 10 µm. **(E)** Confocal images of myelin (Tg(mbp:EGFP-CAAX)) and neuronal cell bodies (Tg(NBT:DsRed)) immediately post 2 days of treatment with 5 mM mtz. Asterisks indicate myelin swellings. Scale bar = 10 µm. **(F)** Confocal images of an individual oligodendrocyte from an animal treated for 1 day with 5 mM mtz at 4 days post-fertilisation (dpf), imaged over time 0 days (5 dpf), 2 days (6 dpf), 4 days (8 dpf) and 5 days (9 dpf) post-mtz treatment where the cell degenerates. Yellow arrows indicate cell body location. Scale bar = 10 µm. dpt = days post-treatment. **(G)** Confocal images of an individual oligodendrocyte from an animal treated for 1 day with 5 mM mtz at 4 dpf, imaged over time 1 day, 3 days and 4 days post-mtz treatment where the cell survives and recovers. Scale bar = 10 µm. dpt = days post-treatment. **(H)** Quantification of number of oligodendrocytes that degenerated or survived following treatment with 5 mM mtz (out of 39 cells from 34 animals). **(I)** Confocal images of individual myelin sheaths from animals treated for 1 day with 5 mM mtz, imaged over time where the swelling recovers or decreases in size. Asterisks indicate location of swellings. Scale bar = 10 µm. dpt = days post-treatment. **(J)** Confocal images of individual myelin sheaths from animals treated for 1 day with 5 mM mtz, imaged over time where the swelling increases in size. Asterisks indicate location of swellings. Scale bar = 10 µm. dpt = days post-treatment. **(K)** Quantification of number of swellings that over a 4-5 day period increased in size, remained stable or decreased in size/recovered following 1-2 days of treatment with mtz (swellings tracked in 16 cells from 14 animals). **(L)** Schematic of experimental timings for imaging myelin swellings in individual oligodendrocytes in the zebrafish Tg(mbp:TRPV1-tagRFPt) model. **(M)** Confocal images of single myelin sheaths labelled with mbp:EGFP-CAAX followed over time in the dorsal spinal cord in Tg(mbp:TRPV1-tagRFPt) animals, imaged pre-treatment, immediately post 2 hours of treatment with vehicle or 5 µM csn, and 1 day later. Scale bar = 10 µm. hpt = hours post-treatment. dpt = days post-treatment. **(N)** Confocal images of myelin sheaths of individually labelled Mauthner-myelinating oligodendrocytes from animals treated for 2 hours with 5 µM csn, imaged over time immediately post-treatment and 1 dpt. Asterisks indicate location of swellings. Yellow arrows indicate cell body location. Scale bars = 10 µm. hpt = hours post-treatment. dpt = days post-treatment.

To study early stages of myelin damage *in vivo* over time, we carried out live imaging in our zebrafish models of demyelination. We first employed the model in which damage to oligodendrocytes can be induced by treatment of Tg(mbp:mCherry-NTR) zebrafish with mtz (21) (Methods). When individual fluorescently labelled oligodendrocytes located in the spinal cord were imaged after 1-2 days of treatment with mtz, extensive swelling of myelin was already evident (Fig. 1A-C). Swelling occurred at multiple sites along the same sheath (Fig. 1B, Fig. S2A). In line with electron microscopy-based observations, co-labelling of myelin with axons and neurons indicated that myelin swelling was not associated with corresponding swelling of the underlying axon or ectopic myelination of neuronal cell bodies (Fig. 1D-E, Fig. S2B, Movie S1). In addition, we observed myelin swelling when we induced damage with mtz at later larval stages indicating that myelin swelling is also characteristic of damage at more advanced stages (Fig. S2C).

To study myelin damage in more detail, we followed 39 individually labelled oligodendrocytes for 4-7 days following the induction of damage. Of these, 41% subsequently underwent myelin loss and degenerated (Fig.1F-H). Degenerating cells were characterized by fragmented or completely lost myelin sheaths (Fig. 1F, S2D). In 43% of the surviving cells (10 out of 23 oligodendrocytes where we could clearly track individual sheaths over time), myelin swelling appeared to remodel: the swelling either resolved completely (Fig. 1I) or decreased in size over time (Fig. 1G, K, Fig. S2A). In addition, while some swellings increased in size over time, others remained stable throughout this observation period (Fig. 1J, K). In a separate experiment, we followed oligodendrocytes for a longer period of time and found instances where swellings remained resolved even 10 days after the induction of myelin damage (Fig. S2E). Together, our data indicate that myelin sheaths can withstand swelling associated with oligodendrocyte damage and that myelin swellings can resolve over time.

To investigate if myelin sheaths can withstand damage and subsequently remodel in a distinct model, we used Tg(mbp:TRPV1-tagRFPt) zebrafish, in which myelin damage and loss is induced upon treatment of animals with csn (15). Following the application of 10 µM csn, we observed that sheaths exhibited myelin swelling and loss, but over a much shorter timescale compared to the Tg(mbp:mCherry-NTR) model (1-2 hours vs days) (Fig. 1L, S3A and B). In some cases, sheaths were already fragmented shortly after treatment (Fig S3B). As in the previous model, co-labelling indicated that myelin swelling was not associated with corresponding changes to axonal morphology (Fig. S3C). Of myelin sheaths that persisted through 10 µM csn-induced damage, some did indeed exhibit the capacity to remodel (Fig. S4A).

As 10 µM csn treatment caused such severe myelin loss, we treated Tg(mbp:TRPV1-tagRFPt) animals with 5 µM csn for 2 hours, through which more myelin sheaths can persist (Fig. S3D). Tracking individual myelin sheaths in this condition revealed many cases in which sheaths exhibited swelling and disruption that subsequently resolved by 1d post-treatment (Fig. 1M, N, Fig. S4B). Indeed, individual sheaths generally maintained their length compared to controls, and in some cases elongated over time (Fig. S4B, D, Fig. 1N). Imaging at multiple timepoints over the course of a day following treatment with 5 µM csn confirmed that sheaths were not simply rapidly lost and replaced, underscoring that remodelling of sheath morphology occurs following myelin damage (Fig. S4C). As the individual myelin swellings were not as large in this model as the NTR-based model, we developed a machine-learning based method to automatically quantify the disruption to myelin, which we termed ‘disorder’ (Fig. S5 and Methods). When we imaged large, isolated myelin sheaths over time following treatment with 5 µM csn, we saw that disorder was increased compared to vehicle-treated controls, immediately after the 2 hour treatment (Fig. S4E). By 1 day post treatment, this value had reduced in 75% of the myelin sheaths followed over time, indicative of dynamic remodelling of sheath morphology (9/12 sheaths, Fig. S4F). Having seen that myelin swelling could resolve, we next wanted to investigate mechanisms that might influence this early feature of myelin damage.

### Neuronal activity exacerbates myelin swelling

Cell and tissue swelling can be caused by dysregulation of ion and solute homeostasis (23, 24) and recent studies have pointed to the role of myelinating oligodendrocytes in regulating ion, neurotransmitter, and metabolic homeostasis at the axon-myelin interface (25–30). Given that during action potential firing, myelinating oligodendrocytes buffer ions in the extracellular and periaxonal spaces to maintain homeostasis, we asked if increasing neuronal activity would exacerbate myelin swelling.

**Fig. 2:**
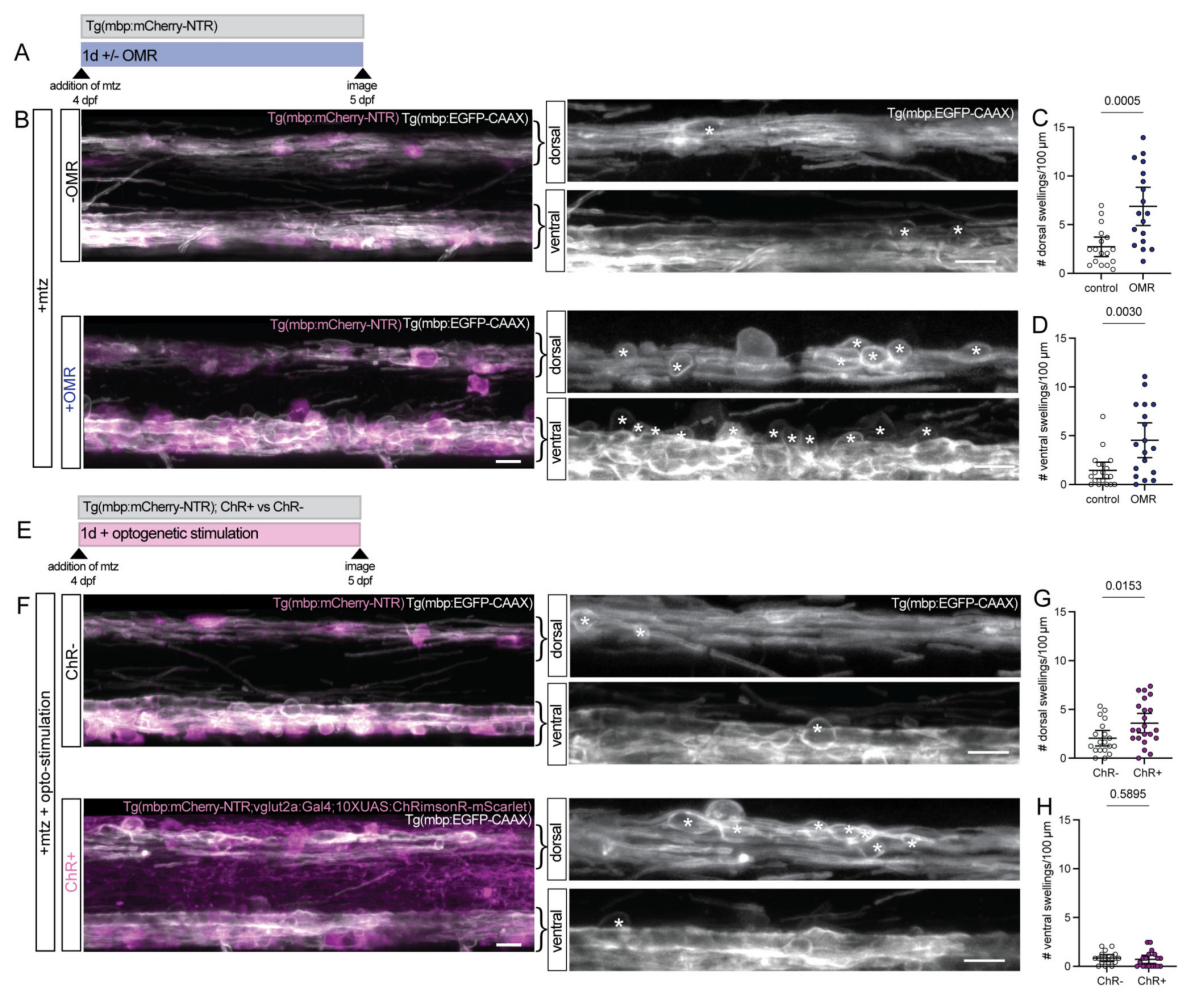
Neuronal activity exacerbates myelin swelling in zebrafish **(A)** Schematic of experimental timings for stimulating swimming activity in zebrafish using the optomotor response (OMR) in the Tg(mbp:mCherry-NTR) model. **(B)** Confocal images of myelin (grey) and oligodendrocytes (magenta) in the dorsal and ventral spinal cord in Tg(mbp:EGFP-CAAX; mbp:mCherry-NTR) animals at 5 dpf following 1 day of treatment with mtz, +/- OMR. Asterisks = myelin swellings. Scale bars = 10 μm. **(C)** Quantification of the number of swellings in the dorsal spinal cord at 5 dpf following 1 day of treatment with mtz, +/- OMR. Data are normalised to 100 µm length of spinal cord. Unpaired t-test with Welch’s correction. Error bars are mean ± 95% CI. **(D)** Quantification of the number of swellings visible above the ventral spinal cord at 5 dpf following 1 day of treatment with mtz, +/- OMR. Data are normalised to 100 µm length of spinal cord. Unpaired t-test with Welch’s correction. Error bars are mean ± 95% CI. **(E)** Schematic of experimental timings for optogenetic stimulation of ChR-expressing glutamatergic neurons in the Tg(mbp:mCherry-NTR) model. **(F)** Confocal images of myelin (grey) and ChR+ glutamatergic neurons and oligodendrocytes (magenta) in the dorsal and ventral spinal cord in Tg(mbp:EGFP-CAAX; mbp:mCherry-NTR) animals at 5 dpf following 1 day of treatment with mtz and optogenetic stimulation. Asterisks = myelin swellings. Scale bars = 10 μm. **(G)** Quantification of the number of swellings in the dorsal spinal cord at 5 dpf following 1 day of treatment with mtz and optogenetic stimulation. Data are normalised to 100 µm length of spinal cord. Unpaired t-test with Welch’s correction. Error bars are mean ± 95% CI. **(H)** Quantification of the number of swellings counted above the ventral spinal cord at 5 dpf following 1 day of treatment with mtz and optogenetic stimulation. Data are normalised to 100 µm length of spinal cord. Unpaired t-test with Welch’s correction. Error bars are mean ± 95% CI.

To assess how neuronal activity influences early myelin pathology, we first increased the physiological swimming activity of zebrafish. We took advantage of the fact that zebrafish exhibit a stereotyped optomotor response (OMR) to stabilise their perceived position in water (31, 32) and developed a virtual reality paradigm (Movie S2, Methods) to stimulate persistent swimming of zebrafish for 24 hours during the induction of myelin damage in our Tg(mbp:mCherry-NTR) model (Fig. 2A). We found that there was an increase in myelin swellings in mtz-treated animals exposed to the OMR stimulus relative to mtz-treated controls (Fig. 2B-D). This indicates that the increased swimming of zebrafish exacerbates myelin swelling, presumably due to increased activity of underlying circuits. Importantly, the OMR stimulus in control animals not undergoing myelin damage did not induce myelin swelling (Fig. S6).

As an independent way to increase neuronal network activity, we used the GABA-A receptor antagonist pentylenetetrazole (ptz), which has previously been shown to increase swimming behaviour of zebrafish when used at low doses (33, 34). In line with OMR-induced swimming, we found that ptz treatment for 1 day alongside mtz increased the amount of disordered myelin and the number of myelin swellings compared to mtz only, whereas ptz-treatment alone did not induce myelin damage (Fig. S7A-D). To test whether increased activity exacerbated myelin damage in the second zebrafish demyelination model, we treated Tg(mbp:TRPV1-tagRFPt) zebrafish with 5 µM csn with either ptz or vehicle for 1 day. We found that the presence of ptz in csn treated animals increased myelin disorder relative to csn-only controls (Fig. S7E-G). Treatment with ptz also increased demyelination along the length of the anatomically-identifiable Mauthner axon (Fig. S7H-J), indicating that myelin damage was exacerbated by stimulating network activity in both zebrafish models.

To test the hypothesis that increasing neuronal activity *per se* influences myelin swelling, we performed optogenetic stimulation experiments. We employed our recently described method (35) for opto-stimulation of neuronal activity in zebrafish larvae over the course of 24 hours. In this case we used animals expressing the red-shifted light-sensitive cation channel ChRimsonR (ChR) under the control of a vglut2a driver expressed in glutamatergic neurons as our experimental group and animals that did not express ChR as controls. We induced myelin damage in ChR-expressing and non-expressing Tg(mbp:mCherry-NTR) animals with mtz, and exposed both groups to light stimulation over 24 hours (Methods). We imaged animals after 1 day and found increased myelin swellings in ChR+ animals relative to ChR-animals (Fig 2E-H). This indicates that optogenetic stimulation increases myelin swelling upon the induction of damage. Together, our studies indicate that neuronal activity represents a risk factor for exacerbated myelin damage, during early stages of demyelination.

### Increased sodium channel activity exacerbates myelin swelling and cell death

**Fig. 3:**
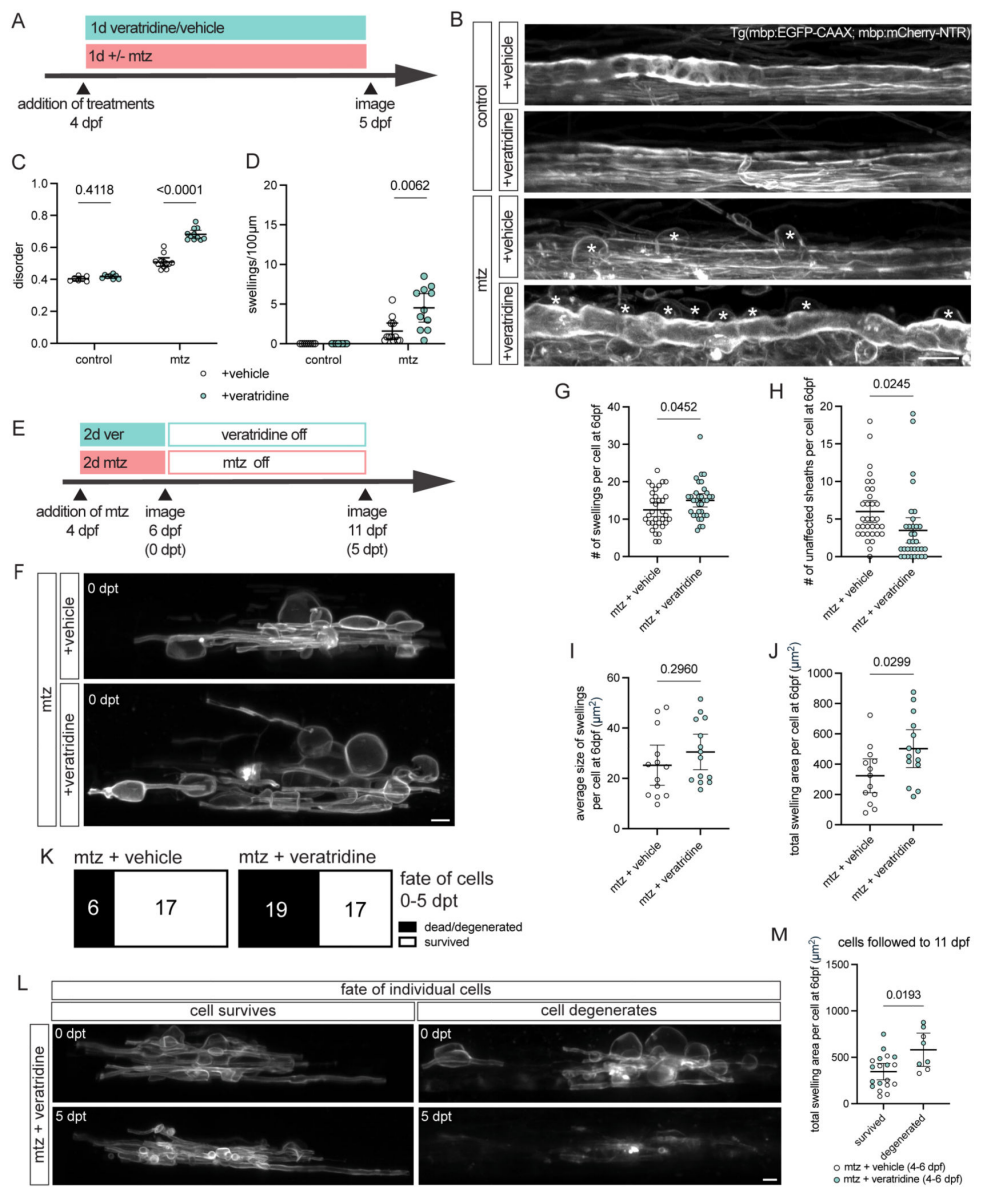
Pharmacologically increasing Na^+^ channel activation exacerbates myelin swelling in zebrafish **(A)** Schematic of experimental set-up for veratridine treatment in the Tg(mbp:mCherry-NTR) model. **(B)** Confocal images of all myelin sheaths in the ventral spinal cord labelled by the transgenic reporter Tg(mbp:EGFP-CAAX) from Tg(mbp:mCherry-NTR)-expressing animals treated with or without mtz for 1 day, with our without treatment with veratridine for 1 day. Asterisks = swellings. Scale bar = 10 µm. **(C)** Quantification of disorder in the ventral spinal cord post-treatment in controls + vehicle (n = 8 animals), controls + veratridine (n = 8 animals), mtz + vehicle (n = 12 animals), or 5 mM mtz + veratridine (n = 11 animals). Two Way ANOVA with Šídák’s multiple comparisons test. Error bars are mean ± 95% CI. **(D)** Quantification of the number of swellings in the ventral spinal cord post-treatment in controls + vehicle (n =8 animals), controls + veratridine (n =8 animals), mtz + vehicle (n = 12 animals), or mtz + veratridine (n = 11 animals). Data are normalised to 100 µm length of spinal cord. Unpaired t-test with Welch’s correction (as values for DMSO-treated groups all = 0). **(E)** Schematic of experimental set-up for veratridine treatment in the Tg(mbp:mCherry-NTR) model for following fate of single oligodendrocytes 5 days post treatment (dpt). Ver = veratridine. dpf = days post fertilisation. **(F)** Confocal images of myelin sheaths in single oligodendrocytes in the ventral spinal cord mosaically labelled with (mbp:EGFP-CAAX) from Tg(mbp:mCherry-NTR)-expressing animals treated with mtz + vehicle or mtz + veratridine immediately post treatment (0 dpt). Scale bar = 5µm. **(G)** Quantification of number of swellings per cell immediately post treatment with mtz + vehicle (n=33) or mtz + veratridine (n=33). One cell per animal. Unpaired two-tailed t-test with Welch’s correction. Error bars are mean ± 95% CI. **(H)** Quantification of number of unaffected myelin sheaths (myelin sheaths with no swellings) per cell immediately post treatment with mtz + vehicle (n=33) or mtz + veratridine (n=33). Unpaired two-tailed t-test with Welch’s correction. Error bars are mean ± 95% CI. **(I)** Quantification of average size of swellings per cell immediately post treatment with mtz + vehicle (n=13) or mtz + veratridine (n=14). Unpaired two-tailed t-test with Welch’s correction. Error bars are mean ± 95% CI. **(J)** Quantification of total swelling area per cell immediately post treatment with mtz + vehicle (n=13) or mtz + veratridine (n=14). Unpaired two-tailed t-test with Welch’s correction. Error bars are mean ± 95% CI. **(K)** Quantification of number of oligodendrocytes that degenerated or survived following treatment with mtz + vehicle (n=23) or mtz + veratridine (n=36). Chi-square test P value = 0.0430. **(L)** Confocal images of myelin sheaths in single oligodendrocytes in the ventral spinal cord followed over time with treatment of mtz + veratridine. Scale bar = 5µm. **(M)** Quantification of total swelling area per cell immediately post treatment with mtz + vehicle (clear datapoints) or mtz + veratridine (blue datapoints), separated into oligodendrocytes that survived (n=19) vs. degenerated (n=8). Unpaired two-tailed t-test with Welch’s correction. Error bars are mean ± 95% CI.

The observation that neuronal activity exacerbates myelin swelling suggests that ion homeostasis at the axon-myelin interface influences myelin damage. Indeed, we previously observed that Na^+^ channel inhibition prevented myelin swelling in the PNS of zebrafish mutant for the gene encoding the ion transporter NKCC1b (23), located at the axon-myelin interface (36). Therefore, we next tested whether Na^+^ channel activity might influence myelin swelling in the CNS. First, we studied Tg(mbp:mCherry-NTR) animals treated with mtz in combination with veratridine, a compound that drives persistent activation of voltage-gated Na^+^ channels (Fig. 3A and B). Addition of veratridine alongside mtz increased the amount of disordered myelin and myelin swellings compared to mtz alone when animals were assessed 1 day into treatment, whereas veratridine did not affect myelin integrity or cause swelling in controls where myelin damage was not induced (Fig. 3C and D).

We next followed the fate of individual oligodendrocytes in animals with increased Na^+^ channel opening following induction of myelin damage. To do so, we imaged single cells after 2 days of treatment with mtz and either veratridine or vehicle control (Fig. 3E). Immediately post-treatment, animals treated with veratridine and mtz exhibited an increased number of myelin swellings per oligodendrocyte compared to mtz-treatment alone (Fig. 3F and G). This was accompanied by a corresponding decrease in the number of unaffected myelin sheaths per oligodendrocyte in veratridine and mtz-treated animals compared to animals treated only with mtz (Fig. 3H). Treatment with veratridine and mtz also resulted in an increase in the total area of swelling per oligodendrocytes compared to mtz-treatment, whereas the average size of individual swellings did not differ (Fig. 3I and J).

To investigate the consequences of the exacerbated myelin swelling caused by veratridine we imaged the same cells again 5 days later. We found that veratridine treatment increased the number of mtz-treated cells that degenerated 5 days after treatment withdrawal (53% of cells in veratridine + mtz treated animals degenerated vs 26% in mtz treated alone Fig. 3K and L). We next assessed the relationship between myelin swelling and cell survival and found that the total area of myelin swelling per oligodendrocyte immediately following treatment was predictive of cell fate 5 days later, whereby cells with a larger total swelling area immediately post-treatment were more likely to have degenerated when assessed 5 days later (Fig. 3M). These analyses indicate that increased Na^+^ channel activity during early stages of myelin damage increases myelin swelling and associated cell death.

### Decreased sodium channel activity mitigates myelin swelling and cell death

**Fig. 4:**
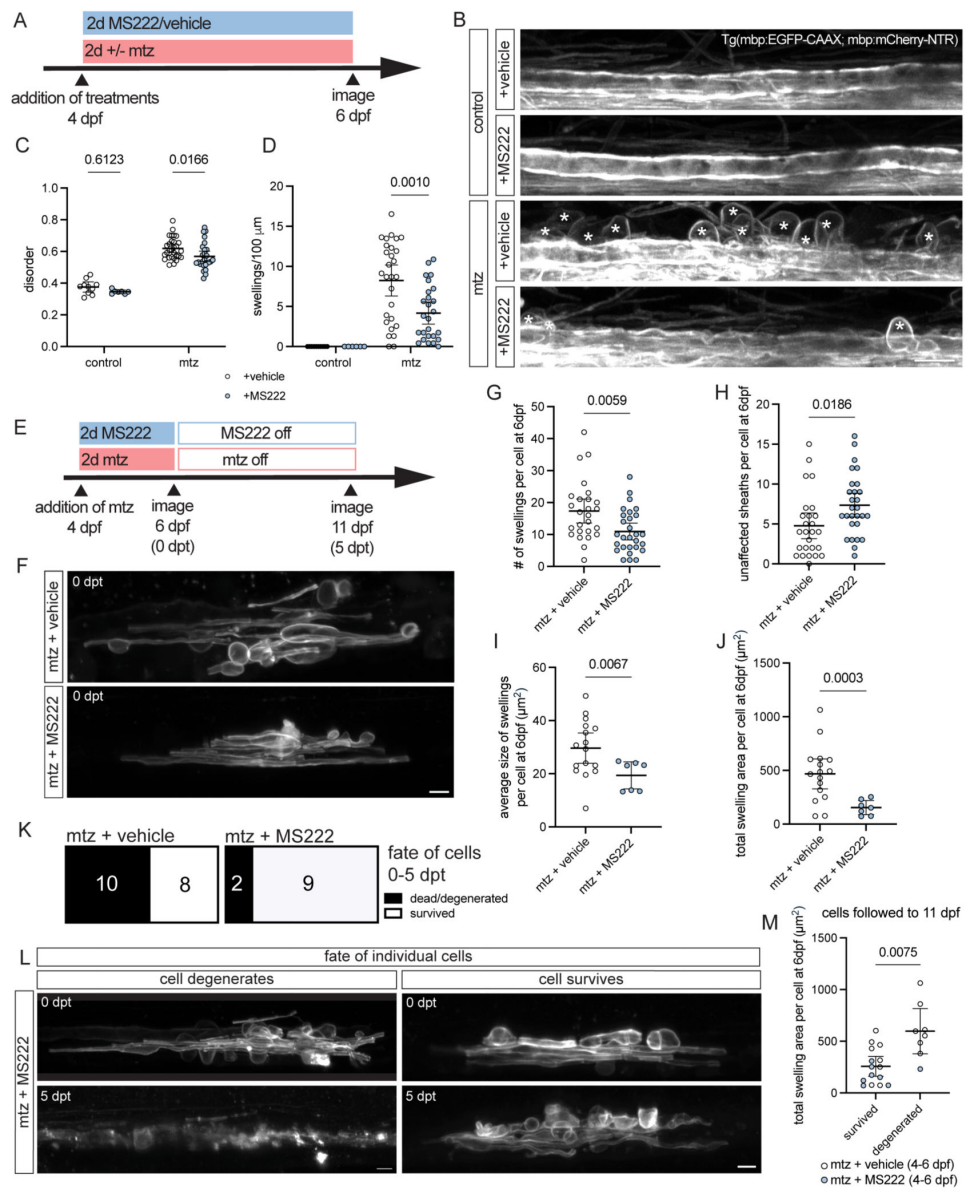
Pharmacological inhibition of voltage-gated Na^+^ channels mitigates myelin swelling in zebrafish **(A)** Schematic of experimental set-up for MS222 treatment in the Tg(mbp:mCherry-NTR) model **(B)** Confocal images of all myelin sheaths in the ventral spinal cord labelled by the transgenic reporter Tg(mbp:EGFP-CAAX) from Tg(mbp:mCherry-NTR)-expressing animals treated with or without 5mM mtz for 2 days, with or without treatment with MS222 for 2 days. Asterisks = swellings. Scale bar = 10 µm. **(C)** Quantification of disorder in the ventral spinal cord post-treatment in controls + vehicle (n = 10 animals), controls + MS222 (n = 7 animals), mtz + vehicle (n = 29 animals), or mtz + MS222 (n = 27 animals). Two Way ANOVA with Šídák’s multiple comparisons test. Error bars are mean ± 95% CI. **(D)** Quantification of the number of swellings in the ventral spinal cord post-treatment in controls + vehicle (n =10 animals), controls + MS222 (n =6 animals), mtz + vehicle (n = 28 animals), or mtz + MS222 (n = 26 animals). Data are normalised to 100 µm length of spinal cord. Unpaired t-test with Welch’s correction (as values for DMSO-treated groups all = 0). **(E)** Schematic of experimental set-up for MS222 treatment in the Tg(mbp:mCherry-NTR) model for following fate of single oligodendrocytes 5 days post treatment (dpt). dpf = days post fertilisation. **(F)** Confocal images of myelin sheaths in single oligodendrocytes in the ventral spinal cord mosaically labelled with (mbp:EGFP-CAAX) from Tg(mbp:mCherry-NTR)-expressing animals treated with mtz + vehicle or mtz + MS222 immediately post treatment (0 dpt). Scale bar = 5µm. **(G)** Quantification of number of swellings per cell immediately post treatment with mtz + vehicle (n=26) or mtz + MS222 (n=28). One cell per animal. Unpaired two-tailed t-test with Welch’s correction. Error bars are mean ± 95% CI. **(H)** Quantification of number of unaffected myelin sheaths (myelin sheaths with no swellings) per cell immediately post treatment with mtz + vehicle (n=26) or mtz + MS222 (n=28). Unpaired two-tailed t-test with Welch’s correction. Error bars are mean ± 95% CI. **(I)** Quantification of average size of swelling per cell immediately post treatment with mtz + vehicle (n=16) or mtz + MS222 (n=7). Unpaired two-tailed t-test with Welch’s correction. Error bars are mean ± 95% CI. **(J)** Quantification of total swelling area per cell immediately post treatment with mtz + vehicle (n=16) or mtz + MS222 (n=7). Unpaired two-tailed t-test with Welch’s correction. Error bars are mean ± 95% CI. **(K)** Quantification of number of oligodendrocytes that degenerated or survived following treatment with mtz + vehicle (n=18) or mtz + MS222 (n=11). Chi-square test P value = 0.0474. **(L)** Confocal images of myelin sheaths in single oligodendrocytes in the ventral spinal cord followed over time with treatment of mtz + MS222. Scale bar = 5µm. **(M)** Quantification of total swelling area per cell immediately post treatment with mtz + vehicle (clear datapoints) or mtz + MS222 (blue datapoints), separated into oligodendrocytes that survived (n=15) vs. degenerated (n=8). Unpaired two-tailed t-test with Welch’s correction. Error bars are mean ± 95% CI.

To test if reducing Na^+^ channel activation might mitigate myelin damage, we first assessed phenytoin, a compound which binds to inactivated Na^+^ channels, blocking the persistent Na^+^ current (37), which has been shown to be neuroprotective in preclinical models of MS (38) and in patients with optic neuritis (39). Treatment of Tg(mbp:mCherry-NTR) animals with phenytoin and mtz decreased both the amount of disordered myelin and the number of swellings in the spinal cord after 2 days compared to mtz treatment alone (Fig. S8). However, phenytoin only acts to limit excessive Na^+^ current, and in addition to modulating Na^+^ channels may bind other targets such as voltage-gated Ca^2+^ channels (40, 41). Therefore, we turned to using MS222, a voltage-gated Na^+^ channel blocker extensively used to anaesthetise zebrafish. We found that treatment with MS222 and mtz also reduced both myelin disorder and myelin swelling relative to mtz alone in Tg(mbp:mCherry-NTR) animals. In non-demyelinating controls, MS222 did not disrupt myelin morphology (Fig. 4A-D).

We next followed the fate of individual oligodendrocytes in animals with decreased neuronal activity. We treated animals with mtz and MS222 or mtz alone for 2 days and then imaged single cells immediately post treatment (Fig. 4E). We found a decrease in the number of myelin swellings per oligodendrocyte in mtz and MS222 treated animals compared to mtz-treatment alone immediately post treatment, and a corresponding increase in the number of unaffected myelin sheaths per oligodendrocyte (Fig. 4F-H). Addition of MS222 and mtz also resulted in a decreased average size and total area of swelling of oligodendrocytes compared to mtz-treated controls (Fig. 4I-J).

To investigate the consequences of the reduced myelin swelling in animals treated with MS222 we imaged the same cells again 5 days later, following treatment withdrawal. Importantly, we found that mtz with MS222 treatment led to a decrease in the number of cells that degenerated compared to mtz alone (18.2% of oligodendrocytes in MS222 + mtz treated animals degenerated vs 55.6% in mtz alone, Fig. 4K and L). We next assessed the relationship between myelin swelling per oligodendrocytes and cell survival and found that the total area of myelin swelling per oligodendrocyte immediately post treatment was predictive of cell survival 5 days later, as noted in our veratridine experiments, with cells exhibiting less swelling more likely to survive (Fig. 4M). These analyses indicate that blocking Na^+^ channel activity during early myelin damage leads to a mitigation of myelin swelling and associated cell degeneration.

### Myelin swelling in a mammalian model of demyelination is activity-dependent

**Fig. 5:**
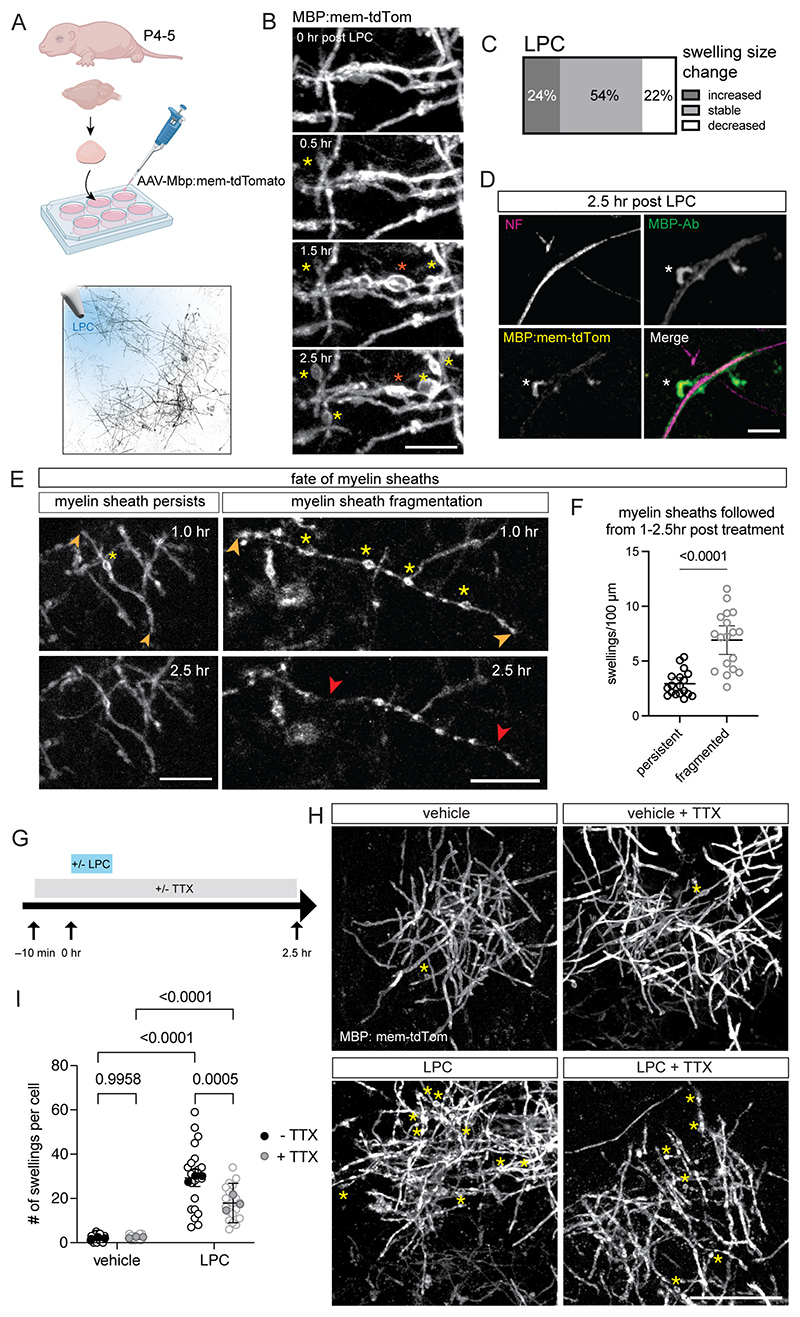
Myelin swelling is dynamic in LPC-treated cortical slices and is mitigated by TTX **(A)** Schematic of experimental set-up for the slice culture preparation and LPC application in ex vivo mouse cortical organotypic slices. **(B)** Exemplar two photon images of myelin swelling after LPC. Yellow asterisks indicate swellings which increase in size, orange asterisks indicate swellings which decreased in size. See also **Movie S3**. Scale bar = 10 µm. **(C)** Quantification of percent of swellings which increased in size, remained stable or reduced following application of LPC, *n* = 72 swellings from *N* = 3 slices. **(D)** *Z*-projected confocal image of a slice fixed 2.5 hours after LPC application showing selective myelin swelling. Marker NF (neurofilament), MBP antibody, AAV-MBP:mem-tdTom and merged image. Scale bar = 5 µm. **(E)** Exemplar two photon images of an internode that persists after LPC induced swelling (left) and an internode which fragments after swelling (right). End of internodes are marked with orange arrows. Yellow asterisks indicate swellings. Red arrows indicate areas of myelin fragmentation. Scale bar, 20 µm. **(F)** Quantification of number of swellings per 100 µm at 1 hour post-LPC treatment that subsequently either persisted or fragmented by 2.5 hours. Unpaired t-test with Welch’s correction. *n* = 18 persistent and *n* = 18 damaged sheaths. Error bars are mean ± 95% CI. **(G)** Schematic of experimental set-up for TTX application in the LPC model. **(H)** Exemplar two photon images of oligodendrocytes from slices treated with vehicle, vehicle+TTX, LPC, LPC+TTX. Yellow asterisks indicate swellings. Scale bar, 50 µm. **(I)** Quantification of swellings/oligodendrocyte at 2.5 hours in slices treated with vehicle, LPC, LPC+TTX, and vehicle+TTX. Two-way ANOVA with Tukey’s multiple comparisons tests. *n* = 26 oligodendrocytes from *N* = 3 slices vehicle treated, *n* = 20 oligodendrocytes from *N* = 3 slices vehicle+TTX treated. *n* = 22 oligodendrocytes from *N* = 3 slices LPC treated, *n* = 21 oligodendrocytes from *N* = 3 slices, LPC+TTX treated. Clear circles represent swelling number per individual oligodendrocyte, filled circles represent average number of swellings per oligodendrocyte per slice. Error bars are mean ± 95% CI.

Next, we investigated the dynamics of myelin swelling in a mammalian model. We employed an *ex vivo* mouse cortical organotypic slice culture model and visualized oligodendrocytes and their myelin sheaths using the viral reporter AAV-Mbp:mem-tdTomato (42) (Methods). Demyelination was induced at 14-21 days in vitro (DIV) by local pressure-injection application of lysolecithin (LPC) via a patch pipette (Fig. 5A). LPC application led to rapid damage of myelin, and the appearance of numerous myelin swellings, which we monitored by live two-photon time-lapse imaging at 15 min intervals for 2.5 hours (Fig. 5B).

We observed that myelin swellings changed dynamically (Fig. 5B, Movie S3) and that myelin sheaths could either persist or fragment over time (Fig. 5E), reminiscent of the swellings seen across our zebrafish models of demyelination. To investigate the dynamic changes to myelin swellings upon LPC treatment, we followed the fate of swellings present 1 hour after treatment for the subsequent 1.5 hours. Over this period, 54% of swellings remained stable, 24% increased in size, whereas 22% decreased in size over time, indicating the potential for myelin swellings to resolve in mammals (Fig. 5B, C). Additionally, post-hoc analysis for an axonal marker (neurofilament, NF), imaged alongside mem-tdTomato, and an antibody to MBP revealed that swellings were selective to the myelin sheaths without changes to the underlying axons (Fig. 5D).

We examined the fate of myelin sheaths with differing degrees of myelin swelling and found that sheaths with more swellings at 1 hour post-treatment were more likely to undergo fragmentation by 2.5 hours post treatment (Fig. 5E, F). This further supports the conclusion that the severity of myelin swelling predicts later sheath degeneration.

Based on our findings in zebrafish, we predicted that myelin swellings might also be influenced by action potentials in mammals. To test this, we treated cortical slices with the highly selective voltage-gated Na^+^ channel blocker, tetrodotoxin (TTX, 1 µM), starting 10 min prior to LPC exposure and continuing during the imaging sessions (Fig. 5G). Indeed, at 2.5 hours post LPC treatment, slices co-treated with TTX had fewer myelin swellings per oligodendrocyte (average 18.19 swellings per cell) compared to LPC treatment alone (average 29.27 swellings per cell). Both vehicle-treated and TTX-only treated slices exhibited very few swellings per oligodendrocyte (Fig. 5I-H).

Together, these results indicate that dynamic swelling of myelin sheaths in response to damage is a conserved feature of early demyelination across both mouse and fish models, and that reducing action potentials can mitigate myelin swelling.

### Myelin swelling is dynamic in human tissue and a characteristic of MS pathology

**Fig. 6:**
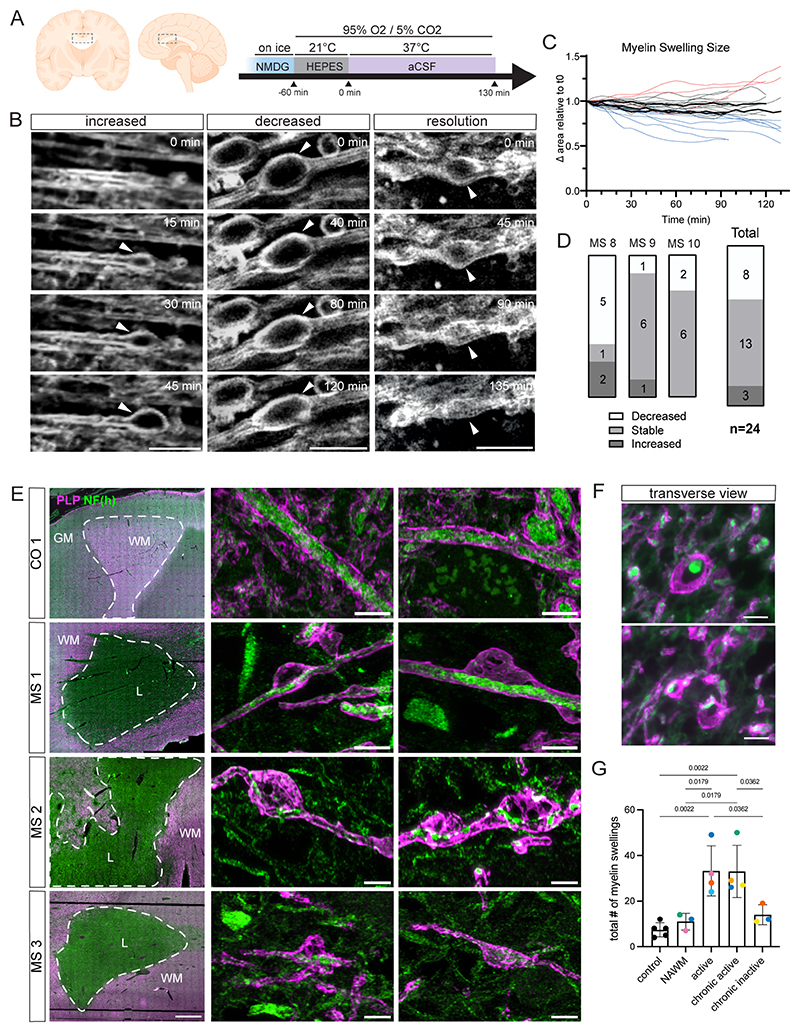
Myelin swelling is dynamic in MS corpus callosum and is present surrounding actively demyelinating lesions in human MS brain **(A)** Coronal (left) and midsagittal (right) view of the corpus callosum excised for THG imaging. Right: schematic of the tissue recovery protocol for live-THG imaging in human corpus callosum sections (Methods). **(B)** Exemplar live THG images of myelin swellings in ex-vivo human corpus callosum sections. Swelling increased (left), swelling decreased (middle) and swelling resolution (right row), marked by arrows. Scale bar = 10 µm. **(C)** Spaghetti plot of the change in area of individual myelin swellings in MS tissue, relative to time zero (t0), with individual swellings followed at 5 minute intervals through approximately 2 hours of THG imaging. Thin lines represent individual swellings and bold lines reflect the mean swelling size per MS case. Colours represent increasing (red), decreasing (blue) or stable swellings (black). **(D)** Quantification of the relative number of swellings which increased in size, remained stable or reduced in MS cases. N=3 cases, in which 8 distinct swellings were followed. Rightmost bar represents the total change in swellings followed across all 3 MS cases. **(E)** Left: Overview images of control and MS brain tissue showing the location of demyelinated lesions (L). Immunofluorescence for PLP/myelin (magenta) and neurofilament/axons (green). GM = grey matter, WM = white matter. Scale bar = 1mm. Middle and right: corresponding confocal images of myelinated axons in control white matter (upper) and examples of perilesional myelin swellings in MS tissue. Scale bars = 5 µm. **(F)** Myelinated axons in MS tissue (MS6) showing swellings in transverse views. Scale bars = 10 µm **(G)** Quantification of the total number of myelin swellings observed in 112,500 µm^2^ control and MS brain tissue split according to NAWM (normal-appearing white matter) and demyelinated lesion type (active, chronic active, and chronic inactive). Colours represent different individuals with MS (n = 6), each datapoint in controls also represents a different individual (n = 5). Mixed-effect model and Holm-Šídák’s multiple comparisons test. Error bars are mean ± 95% CI.

Our documentation of the ability of myelin swellings to resolve highlights a phenomenon of potential relevance to disease. We have previously documented an increase in myelin swellings in people with MS (43–45), however whether myelin swelling retains its dynamic characteristics in a human context remains unclear. In order to assess whether myelin swellings in human MS cases also exhibit dynamic characteristics, we visualised acute physiologically prepared MS human post-mortem corpus callosum sections using Third Harmonic Generation (THG) imaging of myelin (43) (Fig 6A, Methods). We followed myelin swellings by time-lapse THG microscopy in three separate samples over the course of 2 hours. This longitudinal tracking showed that whereas the majority of identified swellings remained stable, some increased in size and others decreased, with some fully resolving (Fig. 6B and C, Movie S4). Although we noted heterogeneity across the three samples, 33% of all identifiable swellings followed over time were observed to decrease (Fig. 6D). This further suggests that the highly dynamic nature of myelin swelling is an evolutionarily conserved phenomenon.

The prevalence of myelin swelling at early stages of demyelination in a range of animal models, and our documentation of swelling in MS tissue, suggests that this may represent a feature of active demyelination in disease. Therefore, we next aimed to further characterise myelin swelling in the context of MS pathology. To do so we assessed whether myelin swelling was present in areas of active demyelination in human MS tissue where axons and myelin were imaged at high resolution. We stained paraffin-embedded sections of post-mortem control and MS brain tissue for proteolipid protein (PLP, myelin) and neurofilament-H (NF(h), axons). We identified demyelinated lesions in MS white matter by a lack of PLP stain (Fig. 6E, (=‘L’) Movie S5, Methods). By confocal microscopy, we observed myelin swellings surrounding axons similar to the swelling of myelin we had observed in zebrafish and rodent models (Fig. 6E and F, Movie S5, further examples in Fig. S9) and previously reported in the literature (44).

These swellings were frequently observed at the demyelinated lesion rim, suggesting they may represent an early stage of ongoing myelin damage. Indeed, quantification of the number of swellings present around lesions revealed that swellings were most frequently observed in the perilesion areas of active and chronic active lesions, which are defined by the presence of myeloid cells involved in active demyelination at their rim. Meanwhile, we observed relatively few myelin swellings in normal-appearing white matter (NAWM), chronic inactive lesions in MS donor tissue, or non-MS donor controls (Fig. 6G) where there is little/no active demyelination. Together these data suggest that myelin swelling may represent an early stage of active demyelination in MS, with the potential to remodel and thus may represent a target for interventions that prevent myelin loss.

## Discussion

Here we report that myelin sheaths in the CNS swell after the onset of myelin damage, that such swelling can prefigure either sheath loss or remodelling and that it is influenced by neuronal activity. The discovery that damaged myelin sheaths can remodel has a range of implications for our understanding of myelin health, its disruption in disease, and potential therapeutic strategies.

Myelin swelling has previously been reported, (5–9), but fixed tissue-based evidence of swelling can potentially reflect artefacts of preservation or processing and reflects only static timepoints, which may have demotivated in-depth dynamic investigations of the phenomenon. Our live imaging reveals that myelin swelling is indeed a dynamic feature of myelin damage across zebrafish, mouse organotypic slice cultures and the human MS corpus callosum. Individual swellings can increase and decrease in size or even resolve over time in the contexts and species studied - a remarkable conserved capability that may allow the nervous system to withstand acute challenges and insults. Future studies will be required to determine whether myelin swelling in these and other contexts is driven by similar mechanisms or varies depending on the type of insult. This will be required to determine the differences and similarities between what we call “myelin swellings” here, which have been referred to using varied terminologies such as ‘vacuoles’ in animal models (5, 6) and human conditions (4), ‘myelinosomes’ described in inflammatory demyelination models (11) and ‘blisters’ in MS (44). Previous studies suggest that there may indeed be mechanistically distinct types of myelin swelling: large myelin swellings, vacuolation, has been associated with splitting at the intraperiod line (extracellular swelling and decompaction between wraps) (46). In contrast, ‘vesiculation’ has been associated with calcium-mediated myelin damage (47–50) that may represent splitting of the major dense line (intracellular swelling and decompaction of the myelin sheath). It is noteworthy that although reducing neuronal activity mitigated myelin swelling in the zebrafish NTR and cortical slice LPC models, treatment with either MS222 or phenytoin did not influence myelin pathology in the zebrafish TRPV1 model (Figure S10), suggesting the possibility that different types of insult may lead to distinct modes of myelin swelling.

Our finding that neuronal activity affects the severity of myelin swelling in the zebrafish NTR and the mouse LPC models, does suggest that there are at least some common mechanisms that regulate myelin swelling, independent of the initial insult. Myelinating oligodendrocytes express ion channels, gap junctions, transporters, and exchangers that play a critical role in buffering of ions, particularly K^+^, released from the axon coincident with the Na^+^-mediated action potentials (24–26, 51, 52). Upon myelin damage, if this buffering becomes disrupted, ongoing or increased activity could lead to K^+^ accumulation in the extracellular space (and potentially also intracellular compartments of myelin). The entry of hydrated K^+^ ions could then trigger a pathological cycle of ion and water accumulation. Unless also disrupted, adhesion of the myelin sheath to the axon (53) could allow ion and water accumulation to drive myelin swelling and even decompaction without sheaths actually being removed from the axon. Therefore, when swelling is slowed down or stopped (for example, with reduced activity), the myelin sheath may be able to remodel simply by clearing water and hydrated ions from myelin. Reconstitution of fluid homeostasis is likely to involve other cell types. Interactions of astrocytes and blood vessels are crucial to fluid homeostasis (49), and disruption to factors expressed in several different cells of the neuron-glial-vascular unit leads to myelin swelling, in both experimental models and in leukodystrophies (54–56). Therefore, identifying mechanisms that prevent or reverse myelin swelling, and that reduce the loss of myelin may be of therapeutic promise.

The reduction or reversal of myelin swelling may also facilitate myelin remodelling, but it is likely that the reconstitution of a robust myelin sheath will require additional mechanisms, beyond the restoration of ion and fluid homeostasis. For example, previous studies have indicated that mature oligodendrocytes can exhibit renewed and robust myelination following induction of growth pathways in adults (17, 57). In addition, carbon-dating techniques indicate that human white matter myelin components are turned over more frequently than oligodendrocytes (58), suggesting formation of new myelin by mature oligodendrocytes. This observation, coupled with the fact that old oligodendrocytes predominate in areas of presumptive remyelination in MS, previously led to the contention that oligodendrocytes that survive myelin sheath loss can form new myelin sheaths (59). However, subsequent studies indicated that the potential of oligodendrocytes that survive loss of myelin to generate new sheaths is limited (14–16). Our findings may resolve this apparent discrepancy. If myelin in MS can be damaged, but not necessarily lost, it could be that myelin remodelling explains the new generation of myelin components, without concomitant generation of new cells, or entirely new sheaths.

The fact that myelin can swell and resolve also provokes reinterpretation of additional studies of changes to myelin in health and disease. For example, it was recently put forward that acute demyelination (over the course of days) and subsequent remyelination (over the course of weeks) can occur following marathon running (60). However, an alternative possibility is that these changes may instead reflect myelin swelling, but not loss, with subsequent myelin remodelling and not necessarily remyelination. Distinguishing between myelin swelling, loss, remodelling and remyelination in living people will require adapting advanced imaging measures, and will be important for assessing myelin health across the life-course as well as in disease.

Preventing or limiting myelin swelling and/or promoting myelin remodelling may represent new therapeutic targets in diseases with damage to or loss of myelin. However, we have much to learn. For instance, our data indicate that more severe myelin swelling is predictive of myelin sheath fragmentation in rodent slice cultures and oligodendrocyte loss in zebrafish. Although it is intuitive that uncontrolled myelin swelling could lead to fragmentation of myelin and cell loss, how myelin damage leads to myelin loss in different contexts remains to be determined. For example, it is likely that the complex responses of inflammatory and immune cells, such as microglia will also influence the fate of myelin following damage (61–64). The capacity of myelin to withstand damage and to remodel may also intuitively appear beneficial, for example by allowing original myelination patterns to be maintained, but alternatively it is possible that previously damaged and remodelled myelin may be disruptive and that its removal and bona fide myelin regeneration is preferable. To address this, we need to understand how remodelled myelin affects the underlying axon, including (re-)organization of functional domains along the axonal membrane needed for robust action potential conduction and metabolic support for axonal health and integrity. If targeting myelin swelling and remodelling does prove beneficial to axons and neural circuits, it will be important to investigate what diseases this may be relevant to, given the increasing number that exhibit myelin disruption (19, 65, 66).

The discovery of myelin swelling and subsequent remodelling provides a new lens through which to re-examine the effects of drugs that have shown efficacy in preclinical models of demyelinating disease. Perhaps compounds that have demonstrated positive effects in animal models, or even in clinical trials, exert some of their effects on myelin swelling/remodelling. For instance, the compound phenytoin has previously been shown to be protective in animal models of inflammatory demyelination, even protecting against axonal loss: perhaps part of the efficacy of this and other compounds might be due to mitigation of myelin swelling, a consequent reduction of myelin loss, which may, in turn, provide neuroprotection. Although we have much to learn, our identification of a conserved response of myelin sheaths to damage illustrates the resilience and plasticity of oligodendrocytes and myelin, which may provide entry points to the treatment of human disorders.

## Materials and Methods

### Zebrafish lines and maintenance

Zebrafish (*danio rerio*) were maintained under standard conditions at the Queen’s Medical Research Institute BVS Aquatics facility, University of Edinburgh. All experiments were performed according to Home Office regulations under project licenses PP3290955, PP5258250 and 70-8436. Adult zebrafish were kept in a 14/10 hour light/dark cycle. Embryos were maintained in plastic petri-dishes (Fisher Scientific) at 28.5°C in 10 mM HEPES-buffered E3 embryo medium, at a maximum density of 50 eggs per dish, until 3-4 dpf, when they were arrayed into 24-well or 6-well plates. Animals were housed individually when repeat analyses were conducted on the same zebrafish and for optogenetic studies, however in all other experiments 3 larvae per well (in 24-well plates) or multiple larvae per plate were housed together. Larval zebrafish were analysed between 4 dpf and 15 dpf, before sexual differentiation in zebrafish. Throughout text and figures, ‘Tg’ refers to stable, germline-inserted zebrafish transgenic lines. The following transgenic lines were used in this study: Tg(mbp:TRPV1-tagRFPt) (15), Tg(mbp:EGFP-CAAX) (67), Tg(mbp:mCherry-NTR) (21), Tg(NBT:DsRED)(68), Tg(hspGFF62A:Gal4;UAS:membraneScarlet) referred to as Tg(Mauthner:Gal4;UAS:mem-mScarlet) (69–71), Tg(vglut2a:Gal4; 10xUAS:ChRimsonR-mScarlet)(72, 73).

### Single oligodendrocyte labelling

To label individual oligodendrocytes, fertilised eggs were co-injected with 5-10 pg pTol2-mbp:EGFP-CAAX DNA plasmid and tol2 transposase mRNA, at the 1-4 cell stage. Between 3-4 dpf, animals were sorted for fluorescence to identify isolated oligodendrocytes for live imaging.

### Metronidazole induction of demyelination

Metronidazole (Sigma, M1547) was weighed out to prepare a fresh 10 mM stock in 2% DMSO or E3 medium only for every experiment. This 2x stock solution was then added to an equivalent volume of E3 embryo medium containing animal(s) in the wells of a 24/96-well plate or petri dishes, to make a final working concentration of 5 mM mtz in 1% DMSO or 1% DMSO only. Larvae were treated with mtz or vehicle, starting from 4 dpf, at 28.5 °C, except when animals were treated in petri-dishes at 13 dpf. After incubation in mtz, larvae were transferred to a fresh solution of E3 medium.

### Capsaicin induction of demyelination

Stocks of csn (Sigma-Aldrich) were prepared to a concentration of 20 mM in 100% dimethylsulfoxide (DMSO) (Fisher Scientific) and stored at -80 °C in 20 μl aliquots. A fresh aliquot was used for each experiment. Larvae were treated by bath-application for 2 hours with the working concentration of 5 or 10 μM csn in DMSO or vehicle control (1% DMSO) at 28.5 °C, always at 4 dpf. At the end of this 2 hour incubation period, larvae were transferred to fresh E3 medium.

### Zebrafish drug treatments

Drug treatments were performed by bath application. Pentylenetetrazole (ptz, Tocris, #2687) was stored at a stock concentration of 200 mM in H_2_O at -20°C. For experiments, ptz was used at a working concentration of 3 mM for 18 hours, coinciding with application of csn in the case of the Tg(mbp:TRPV1-tagRFPt) model. Ptz was added at 8 hours post initial application of mtz for 16 hours in the case of the Tg(mbp:mCherry-NTR) model. Veratridine (Tocris, #2918) was stored at a stock concentration of 20mM in DMSO at -20°C. For experiments to enhance sodium channel activity, veratridine was used at a concentration of 2.5 µM for 24 hours coinciding with application of mtz, or at a concentration of 1 µM for 48 hours coinciding with application of mtz.

Tricaine methanesulfonate (MS222 in text and figures, Thermo Scientific, # 118000500), was prepared to a stock concentration of 15 mM (in H_2_O + 2% 1 M Tris buffer pH 9) and stored at 4°C. For experiments to reduce sodium channel activity, MS222 was used at a concentration of 0.2 mM, and animals were treated for 48 hours coinciding with application of mtz, with daily medium changes in the Tg(mbp:mCherry-NTR) model. Phenytoin (ApexBio) was stored at a stock concentration of 10 mM in DMSO and used at a working concentration of 80 µM for experiments. Animals were treated for 48 hours coinciding with application of mtz in the Tg(mbp:mCherry-NTR) demyelination model.

### Zebrafish live imaging

Animals were anaesthetised in 0.6 mM MS222 in E3 embryo medium for live imaging. For single timepoint analyses, larvae were positioned laterally on glass coverslips using a forceps, immobilised in 1.3-1.5% low melting-point agarose. Coverslips were mounted over a slide using high-vacuum silicone grease to create a well containing E3 embryo medium and 0.6 mM MS222. Z-stacks were obtained using a Zeiss LSM880 Airyscan confocal microscope using FAST mode, and a 20X or 40X objective (Zeiss Plan-Apochromat 20X dry, numerical aperture = 0.8; Zeiss C-Apochromat 40X water, numerical aperture = 1.2).

All images of myelin, using Tg(mbp:EGFP-CAAX), following drug treatments were taken from a lateral view of the spinal cord, by lining up the bottom of the field of view at the level of the urogenital opening, unless otherwise stated. To repeat image individual oligodendrocytes, larvae were removed from the agarose following image acquisition and returned to E3 embryo medium and checked for signs of impaired health or abnormal swim behaviour. Between imaging sessions zebrafish were returned to the 28.5°C incubator. The exact somite position was noted in the first imaging session and used to orient the imaging window for the subsequent imaging sessions. Other landmarks within the same channel (such as neighbouring cells) or another channel (such as the Mauthner axon/oligodendrocyte cell bodies) were then used as additional references from the previous images to find the exact same cell/location for the following timepoints. This was again double-checked at the analysis stage by cross-referencing images taken from different timepoints to ensure the same cell/region had been captured over time.

For longer term imaging in timelapse experiments, animals were immobilised using the neuromuscular blocker α-bungarotoxin (Tocris) at 4 dpf. α-bungarotoxin was prepared to a stock concentration of 1 mg/mL, and 20 µl aliquots stored at -20°C. For experiments, all embryo medium was first removed from animals in a glass dish before bath application with α-bungarotoxin for 1 minute each and re-immersion in E3 embryo medium. For overnight imaging, immobilised animals were maintained in 1.5% low melting-point agarose in a plastic 50 mm dish containing E3 embryo media. The incubator was set to 28°C. Z-stacks were obtained using FAST mode, and a 20X objective (Zeiss Plan-Apochromat 20X water-dipping, numerical aperture = 1.0), and images acquired every 20 minutes for up to 16 hours. Where imaging was conducted during csn treatment, csn was added to the petri dish by bath application and removed between imaging timepoints. In some cases, animals were then removed from the microscope and re-imaged up to 35 hours post-treatment with csn, ensuring that animal health was still optimal.

### Optomotor-based stimulation of swimming in zebrafish

Tg(mbp:mCherry-NTR); Tg(mbp:EGFP-CAAX) zebrafish were sorted for NTR (mCherry) and GFP expression at 4 dpf and transferred to petri-dishes containing 5 mM mtz. To induce swimming using the OMR, zebrafish were presented with a pattern of black and white bars, 2mm wide moving at a speed of 20mm per second, which stimulates swimming in the direction of the moving bars (Movie S2). Persistent swimming was maintained by alternating the direction of the moving bars at 20 second intervals. Petri-dishes containing animals where the moving bars were obscured from view were used as controls. Zebrafish were maintained in these conditions for 24 hours before being anaesthetised for live imaging of myelin. The moving pattern was configured using custom software available here https://github.com/klathem/Optical-Treadmill.

### Optogenetic-based stimulation of glutamatergic neurons and assessment of myelin swellings

Optogenetic-based stimulation was conducted similarly to previous studies (35). In brief, Tg(mbp:mCherry-NTR; mbp:EGFP-CAAX; Tg(vglut2a:Gal4; 10XUAS:ChRimsonR-mScarlet) (72, 73) zebrafish were screened by fluorescence for expression of NTR (mCherry) and membrane-tethered GFP in oligodendrocytes, and the channel rhodopsin ChR (mScarlet) in neurons. Zebrafish were subsequently arrayed one animal per well into a 96-well plate already containing 5 mM mtz at 4 dpf. Controls (siblings) were animals in the same plate that were opto-stimulated but did not express ChR. To test response to opto-stimulation, swim behaviour was assessed in ChR-expressing animals following light-exposure and those animals exhibiting a reliable response were used for the experiment. All zebrafish in the plate were exposed to light by widefield illumination using a 569 nm LED every 15 minutes for 24 hours with a motorised Zeiss Observer T1 system, as previously described (35). The plate was sealed to prevent evaporation, and zebrafish were maintained at 28.5 °C for the duration of the stimulation. At the end of the long-term stimulation, zebrafish were then anaesthetised and imaged by confocal microscopy on an LSM880 using a 20X objective.

### Zebrafish image analysis

Most image processing and analysis was conducted using Fiji (Version 2.3.0/1.53f). Z-stacks were converted to maximum intensity projections (MIPs), unless otherwise stated. Images were then rotated so that the anterior is always displayed on the left and dorsal is at the top of the image. For display purposes, brightness and contrast have been adjusted in figures, but this was unchanged between conditions prior to any analysis. Prior to any manual analyses images were randomised and the analyser blinded to experimental condition.

Sheath length was measured manually using the segmented line tool in Fiji. Comparison of individual sheaths over time was done by carefully comparing images from different timepoints post-measurements to ensure that the same cell/sheath was being compared. For dorsal oligodendrocytes, only a subset of sheaths from each cell that were isolated and visible at all timepoints were included in analyses. To quantify myelin damage in entire ventral spinal cords following drug treatments in the Tg(mbp:mCherry-NTR) model, the number of myelin swellings that protruded above the ventral spinal cord in a maximum intensity projection spanning a 235 µm section of spinal cord were counted and displayed graphically per 100 µm.

To assess myelin disorder, automated segmentation was performed using a custom pipeline designed using Arivis Vision 4D. Maximum intensity projections of z-stacks of the mbp:EGFP-CAAX reporter were first imported into the software and used to train a machine-learning based algorithm. The overall signal within each image was detected (‘myelin’). Next, object classes were manually defined and categorised into linear, normal-appearing myelin running along the spinal cord (termed ‘ordered’ throughout this study, predominantly composed of regions with more horizontally aligned pixels) and regions of myelin swelling (termed ‘disordered’ throughout this study, predominantly including more vertically aligned pixels). Examples of segmentation are in Fig. S5. The batch analysis module was used to process the images using the pipeline. Data were expressed as the ratio between the amount of disordered:ordered plus disordered pixels detected in the ‘myelin’ compartment and this value per image or animal as specified in figure legends was used for statistics.

To assess % myelination/demyelination of the Mauthner axon from images taken of Tg(mbp:EGFP-CAAX) animals, MIPs were cropped to only include the top edge of the myelin surrounding Mauthner axon (height of 6 µm for each image). The ‘axon trace’(71) tool was used to trace the highest intensity profile along the image and the ‘plot histogram’ function in Fiji with a grey value threshold of 2000 used to then determine % myelination along this trace.

To assess myelin swellings following optogenetic stimulation, z-stacks containing the dorsal half of the spinal cord closest to the objective were scrolled through to manually count individual myelin swellings. Following counting swellings, the red channel of the image was used to exclude any counted that were oligodendrocyte cell bodies. For analyses of ventral spinal cord, swellings that were visible above the anatomically identifiable Mauthner axon were manually counted on maximum intensity projections. All counts were performed using Fiji.

To assess swelling burden in single oligodendrocytes, number of swellings and unaffected sheaths were manually quantified through the z-stacks using cell counter plugin on Fiji. The area of swelling was assessed by manually tracing swellings based on the stack in which they looked largest. The average swelling size is the average area of all swellings per cell, while the total swelling area is the sum of the area of all swellings per cell. We always assessed one cell per animal, unless otherwise stated in the figure legend.

### Zebrafish live imaging statistics and reproducibility

After sorting for fluorescence, for all experiments larvae were randomly assigned to different treatment conditions. During imaging, animals from independent groups were imaged in an alternating pattern to preclude any effects of time on the parameters being assessed. Manual analyses were always conducted blinded to treatment condition where the effects of drug candidates were being assessed in Tg(mbp:EGFP-CAAX) animals, by two independent blinded experimenters. No data points were excluded from analysis due to variability. All data presented in this study are from experiments conducted over multiple clutches (2-8) of zebrafish over multiple days.

Sample sizes are comparable to what has previously been used for zebrafish live imaging studies (15). Unless otherwise stated in the figure legend, N refers to an individual animal, and if multiple cells were analysed per animal values for these were averaged and noted in figure legends. Comparisons of datasets were conducted by parametric statistical analyses unless otherwise stated, using GraphPad Prism GraphPad Software, Inc., San Diego, United States. In graphs, normally distributed data are displayed as mean ± 95% confidence interval (CI) unless otherwise stated. A difference was considered statistically significant when P < 0.05.

### Cortical organotypic slice culture preparation and culturing

To prepare slice cultures we used male and female C57BL/6JRj mice. All animal procedures were performed with the approval from the Royal Netherlands Academy of Arts and Sciences (KNAW) Animal Ethics Committee (DEC) and Central Authority for Scientific Procedures on Animals (CCD, license AVD80100202216329), and overseen by the Animal Welfare Body (IvD, NIN.22.21.02). Cortical organotypic slice cultures were prepared from 4-5 day old mouse pups by anaesthetizing animals via hypothermia followed by decapitation with scissors. The brain was extracted and placed in ice-cold dissection solution consisting of 98% GBSS (in mM) 137 NaCl, 1.5 CaCl_2_, 0.2 KH_2_PO_4_, 0.3 MgSO_4_, 2.7 NaHCO_3_, 5 KCl, 1 MgCl_2_, 0.85 Na_2_HPO_4_, 5.6 D-glucose), 1% (0.1 M stock) kynurenic acid, and 1% (2.5 M stock glucose), sterile filtered with 0.2 µm filtration flasks, and adjusted to pH 7.2 and 320 mOsm. Under a dissection microscope, brains were cut down the midline with a scalpel and then sectioned via McIlwain Tissue Chopper to obtain 300 µm thick coronal slices. Slices were quickly transferred to hydrophilic PTFE membrane inserts (Merck-Millipore, PICMORG50) in 6-well plates and cultured at 35 °C and 5% CO2 in culturing medium consisting of 47.75% MEM (Thermo Fisher Scientific # 11575032), 25% HBSS (Thermo Fisher Scientific # 24020133), 25% heat-inactivated horse serum (Thermo Fisher Scientific # 26050088), 1% (2.5 M stock) D-glucose, and 1.25% (1 M stock) HEPES (Sigma-Aldrich H3375), sterile filtered, and adjusted to pH 7.2 and 320 mOsm. Medium was changed 3 x a week with fresh equilibrated medium. AAV-MBP:mem-Tdtomato was applied directly to slices cultures at 7 DIV to selectively label oligodendrocytes and allow for visualization of the membrane structure of myelin sheaths with the myelin basic protein (MBP)-specific membrane-bound tdTomato fluorophore (42).

### Cortical organotypic slice culture live two-photon imaging

14-21 days in vitro (DIV) cultures were placed in the recording chamber of a two-photon microscope (Femto-3D-RD, Femtonics Inc., Budapest, Hungary), perfused with carbogen-bubbled recording solution (in mM: 125 NaCl, 25 NaHCO_3_, 1.25 NaH_2_PO_4_, 3 KCl, 25 D-Glucose, 2 CaCl_2_, 1 MgCl_2_). TdTomato labelled oligodendrocytes were visualized via a Ti:Sapphire laser (Chameleon Ultra II; Coherent, Inc.) tuned to 1030 nm, cells were imaged using a 1.0 NA 20x lens (Olympus) with a voxel size of 0.3 × 0.3 × 1 µm (X/Y/Z) at 15 min intervals. Image acquisition took place in MES software (Femtonics Inc., version 6.3.7902). Acquired time-lapse images were imported into Fiji (Fiji 64 bit; ImageJ version 1.54p; RRID: SCR_002285) and swellings were manually counted. The SNT plug-in was used to measure internode length (74). All slice culture data presented in this study were conducted on 3 slices per condition (biological replicates), derived from 2-3 animals across separate culturing preparations. N refers to individual slices and *n* refers to single oligodendrocytes (technical replicates) unless otherwise stated. All statistical comparisons were made using GraphPad Prism and all data in graphs are displayed as ± 95% CI unless otherwise stated.

### LPC induction of demyelination

LPC (Sigma #L4129) was prepared to a concentration of 10 mg/ml in a carbogen bubbled recording solution and loaded into a glass patch pipette. Brief (several seconds) positive pressure was applied to the pipette to locally perfuse the region of imaging with LPC. The final bath concentration will be a magnitude of order lower once LPC has diffused into solution (~1.0 mg/ml). Control imaging sessions used pressure applications with pipettes containing recording solution without LPC.

### TTX treatment in cortical organotypic slice cultures

TTX (Bio-Techne #1069) was dissolved in water to a final stock concentration of 5 mM and stored at –20 °C until use. Fresh aliquots were used to make a working concentration of carbogen bubbled recording solution containing 1 µM TTX for each experiment. Organotypic slice cultures were treated with 1 µM TTX for 10 min prior to induction of demyelination via LPC and through the duration of imaging.

### Immunohistochemistry and confocal imaging of cortical organotypic slice cultures

Cortical organotypic slice cultures were removed from the imaging chamber following imaging sessions and fixed with 4% PFA in PBS for 20 min followed by 3 × 10 min washes in PBS. Slices were then blocked for 2 hr in PBS containing 0.5% Triton X-100 and 10% normal goat serum. Next, slices were incubated 0.25% Triton X-100 and 5% normal goat serum in PBS containing primary antibodies, overnight at room temperature while shaking. Primary antibodies used: Guinea pig polyclonal antibody to RFP (Synaptic Systems, Cat: 390 004, RRID: AB_2737052, 1:500), Mouse monoclonal antibody to pan-axonal neurofilament marker SMI-312 (Eurogentec Cat: SMI 312P 050, 1:6000), Chicken polyclonal IgY antibody to MBP (Aves Labs, Cat: MBP, RRID: AB_2313550, 1:200). The following day, slices washed 3 × 10 min in PBS.

After washing, slices were incubated in PBS containing secondary antibodies for 2 h at room temperature while shaking and protected from light. Slices were finally washed in PBS 3 × 10 min before mounting with Vectashield mounting medium containing DAPI (Vector Laboratories #H-200). Secondary antibodies used: goat anti-mouse IgG, IgM (H+L) secondary antibody, Alexa Fluor 488 (A10684, RRID: AB_2534064). goat anti-guinea pig IgG (H+L) highly cross-adsorbed secondary antibody, Alexa Fluor 594 (A11076, RRID: AB_141930). Goat anti-chicken IgY (H+L) secondary antibody, Alexa Fluor 647 (A21449, RRID: AB_2535866).

Stained slices were imaged on a Leica SP8 confocal microscope using a 40x 1.3 NA oil-immersion lens and running LASX (3.5.7). Images were acquired using sequential scans of individual channels using step sizes of 0.299 µm along the z-axis and at a 2048 × 2048 pixel resolution. Images were imported in Fiji and maximum projection function was used to generated for use in Figure 5.

### Preparation of human tissue slices from MS donors

For live imaging experiments we used acute slices from post-mortem donors, acquired from the Netherlands Brain Bank (www.brainbank.nl, 2009/148). The whole study was performed in strict compliance with ethical requirements of the Amsterdam University medical centers/VU medical centrum, Amsterdam and the Netherlands Code of Conduct for Research Integrity and the Declaration of Helsinki. Informed consent was asked to the donors. For tissue preparation, we sectioned corpus callosum brain tissue (selecting an area between the genu and the trunk of the corpus callosum) from 3 MS donors with short post-mortem delay (Table S2), using a protocol previously published (43). In short, immediately after resection samples were cooled for ten minutes in ice-cold N-methyl-D-glucamine (NMDG) buffer comprising: 93 mM NMDG, 2.5 mM KCl, 1.2 mM NaH_2_PO_4_, 20 mM HEPES, 12 mM N-acetyl-L-cysteine (NAC), 5 mM sodium ascorbate, 3 mM sodium pyruvate, 10 mM MgSO_4_, 30 mM NaHCO_3_, and 25 mM glucose; pH 7.4 before being cut to create flat tissue surfaces. Protective slice recovery was performed in oxygenated NMDG at room temperature for three minutes with carbogen (95% O2/5% CO_2_, at a 0.1 L/min flow rate), followed by a 60-minute oxygenated incubation at room temperature in 50 mL of HEPES holding solution, containing: 92 mM NaCl, 2.5 mM KCl, 1.2 mM NaH_2_PO_4_, 20 mM HEPES, 1 mM NAC, 5 mM sodium ascorbate, 3 mM sodium pyruvate, 0.5 mM MgSO_4_, 1 mM CaCl_2_, 30 mM NaHCO_3_, and 25 mM glucose; pH adjusted to 7.4. Subsequently, each slice was placed on a #1.5H glass coverslip (µ-Dish 35 mm, high glass bottom, ibidi, Gräfelfing, Germany) for live-cell recording, secured by a custom-cut sponge or harp to reduce tissue drift during imaging.

### Third harmonic generation microscopy

To image the samples using THG microscopy, sample holders with prepared brain slices were transferred to a stage-top incubation chamber (H301-PRIOR-H117, OKOLAB S.R.L.), maintained at a constant temperature of 37 °C under continuous carbogen-95% O_2_/5% CO_2_ flow). For live-cell imaging, continuous perfusion with artificial cerebrospinal fluid (aCSF) was conducted at a rate of 1 mL/min using peristaltic pumps (ISM832C, ISMATEC, Cole-Parmer GmbH). The aCSF was comprised of 125 mM NaCl, 15 mM KCl, 2 mM CaCl, 1.25 mM NaH_2_PO_4_, 1 mM MgSO_4_, 26 mM NaHCO_3_, 10 mM C_6_H_12_O_6_; with a pH maintained at 7.4. Time-lapse recordings were conducted with a 200 × 200 µm or 400 × 400 µm FOV within a 1000 × 1000-pixel image at an acquisition speed of one frame per 1.8 seconds. THG images were generated every 5 minutes for a total of 1.5~2 hours. Recordings, including THG time-lapsed z-stacks, were initially saved as 8-bit grayscale BMP files and THG channels were automatically processed for histogram normalization before being converted into TIFF stacks using the Fiji software suite for further data analysis.

### Swelling imaging and analysis of human tissue slices

Images were imported in Fiji and rotated to have axons oriented horizontally and corrected for XY drift. Swellings with a detectable outline were included for tracking longitudinally for 1.5 hours. A custom-made ImageJ macro was used to correct potential drift in Z throughout the time-series. Manually, outlines around the myelin swellings were drawn with the Fiji polygon function to measure swelling areas. The area of swelling was assessed by manually tracing swellings based on the stack in which they looked largest. LOESS smoothing was applied to spaghetti plots.

### Human post-mortem brain tissue histology

Post-mortem brain tissue from MS patients and non-neurological controls were provided by a UK prospective donor scheme with full ethical approval from the UK Multiple Sclerosis Society Tissue Bank (MREC/02/2/39) and from the MRC-Edinburgh Brain Bank (16/ES/0084). MS diagnosis was confirmed by neuropathological means by F. Roncaroli (Imperial College London) and Prof. Colin Smith (Centre for Clinical Brain Sciences, Centre for Comparative Pathology, Edinburgh) with no signs of confounding neurodegenerative diseases and clinical history was provided by R. Nicholas (Imperial College London) and Prof. Colin Smith. Table S1 includes anonymised details on samples used.

For histological analysis, 4 μm sequential sections from paraffin tissue blocks of 2 cm × 2 cm × 1 cm were stored at room temperature. All brain donor samples were from cortical hemisphere white matter. The MS samples chosen had confirmed white matter demyelinated lesions from primary motor, frontal, or periventricular white matter regions and non-MS samples were from primary motor and frontal white matter. The MS samples chosen had confirmed white matter demyelinated lesions. Overall, 7 male and 6 female samples were used (controls: 4 male and 2 female samples, MS: 3 male and 4 female samples).

### Human post-mortem brain tissue immunofluorescent staining

Paraffin sections were rehydrated and microwaved for 15 min in Vector Unmasking Solution for antigen retrieval (H-3300, Vector). For immunofluorescence, sections were incubated with Autofluorescent Eliminator Reagent (2160, MERCK-Millipore) for 1 min and briefly washed in 3% hydrogen peroxide after antigen retrieval, then washed and blocked for 1 hour with 10% normal horse serum, 0.5% Triton-X in TBS. Primary antibodies were diluted in serum block and incubated overnight at 4 °C in a humidified chamber. Primary antibodies used: rabbit recombinant monoclonal IgG antibody to PLP (Clone EPR23504-106, AB254363, Abcam, 1:100) and chicken polyclonal IgY antibody to NF-H (Clone Poly28226, Biolegend, Cat: 822601, RRID: AB_2564859, 1:100). The next day, sections were incubated with Alexa Fluor secondary antibodies (Thermo Fisher Scientific, 1:1000) for 1 hour at room temperature and counterstained with Hoechst or DAPI for nuclear visualisation. Secondary antibodies used: goat anti-chicken IgY secondary antibody, Alexa Fluor 647 (A21449, RRID: AB_2535866), donkey anti-rabbit IgG highly cross-absorbed secondary antibody, Alexa Fluor 568 (A10042, RRID: AB_2534017), donkey anti-chicken IgY (H+L) highly cross-absorbed secondary antibody, Alexa Fluor 488 conjugate, A78948, RRID: AB_2921070. All slides were mounted using SouthernBiotech Fluormount-G slide mounting medium (Cambridge Bioscience, #0100-01).

### Human post-mortem brain tissue imaging

Entire sections were imaged on a Zeiss Axioscan Slidescanner with a 40x objective (Zeiss Plan-Apochromat 40x, numerical aperture = 0.95) and lesions were identified by a lack of PLP stain using Zeiss ZEN lite imaging software. High-resolution images were obtained using an LSM880 confocal microscope using a 63x objective (Zeiss Plan-Apochromat 63x oil, numerical aperture = 1.4). Z-stacks were acquired with an optimal z-step according to the experiment, all exemplar images are maximum intensity projections.

### Human post-mortem brain tissue quantification

Analysis was done using QuPath version 0.4.4 and Fiji ImageJ 64-bit version 2.14.0/1.54f software. Lesion borders were drawn on QuPath based on distinct areas of demyelination as seen by the PLP stain. The perilesional area was created using a 200 µm border from the lesion edge, and within this area we randomly selected 5 locations with longitudinally tracking axons (to ensure swellings could be identified) and created 150 µm x 150µm boxes to count the number of swellings, see Fig. S11. For NAWM and control non-MS tissue, we randomly selected 5 apparently normally myelinated locations by PLP staining. Demyelinated lesion types were defined based on standard pathology classification into active, chronic active, and chronic inactive lesion types (75) using number and location of infiltrating immune cells with sharpness of the demyelinated lesion edge. Following quantification of 5 locations, we summed the total number of myelin swellings per lesion type/NAWM/control tissue (112,500 µm^2^). Of note, the 4 µm thick sections do not allow reconstruction of whole oligodendrocyte morphology, so data is displayed as number of swellings per area.

### Genetic ablation of Myrf and tissue processing

Mice were maintained in the Oregon Health & Science University animal facility in pathogen-free conditions on a 12-hour light/dark cycle. Myrf floxed mice (76) on a C57BL/6 background were crossed to the Plp1-CreERT mouse line (77) (Jax line 005975) to generate Myrf^Fl/Fl^; Plp1-CreERT^+^ (Myrf^ΔiPLP1^) mice and Myrf^Fl/Fl^; CreERT^-^ control littermates. At eight weeks of age all mice were treated with tamoxifen (T5648, Sigma, 100mg/kg i.p. for five consecutive days) to induce recombination of the floxed *Myrf* allele and subsequent demyelination in CreERT^+^ animals. At eight weeks post tamoxifen mice were deeply anesthetized with ketamine (400 mg/kg) and xylazine (60 mg/kg) and transcardially perfused with 10 mL of phosphate buffered saline (PBS) followed by 40mL of freshly prepared 4% paraformaldehyde in PBS. All animal procedures were performed in accordance with, and approved by, the Institutional Animal Care and Use Committee of OHSU.

Tissue was processed for electron microscopy as previously described (20, 78). Dissected optic nerves were postfixed overnight in 2% paraformaldehyde and 2% glutaraldehyde in PBS before being stored for one to two weeks in 1.5% paraformaldehyde, 1.5% glutaraldehyde, 50mM sucrose, 22.5mM CaCl_2_ in 0.1M cacodylate buffer. Nerves were infiltrated with 2% osmium tetroxide (19190, Electron Microscopy Sciences) and 1.5% potassium ferrocyanide (25154-20, Electron Microscopy Sciences) using a Biowave Pro+ microwave (Ted Pella), stained with 0.5% uranyl acetate (22400, Electron Microscopy Sciences), dehydrated in successive grades of acetone and embedded in EMbed 812 (14120, Electron Microscopy Sciences). 60nm sections two millimetres from the optic chiasm were mounted on copper grids (T400-Cu, Electron Microscopy Sciences) and counter stained with 5% Uranyl Acetate followed by Reynold’s Lead Citrate (80 mM Pb(NO_3_)_2_ 17900-25, Electron Microscopy Sciences) and 120mM Sodium Citrate (21140, Electron Microscopy Sciences). Grids were imaged at 4800x on a FEI Tecnai T12 transmission electron microscope with a 16 Mpx camera (Advanced Microscopy Techniques Corp). Quantification of large vacuoles/swellings following *Myrf* ablation was done manually on sections prepared from Myrf^ΔiPlp1^ (average area analysed per animal =28372 um^2^) and Myrf^fl/fl^ animals (average area analysed per animal =23605 um^2^) 10 weeks post-tamoxifen.

### Zebrafish electron microscopy

Zebrafish samples were prepared for electron microscopy as previously described(15, 21, 79). Briefly, primary fixation of terminally anaesthetised zebrafish was performed by immersion in 4% paraformaldehyde (Agar Scientific, #R1026, EM grade) + 2% glutaraldehyde (Agar Scientific, #R1020, EM grade) in 0.1 M sodium cacodylate buffer (pH 7.4), with microwave stimulation. Following overnight incubation, secondary fixation consisted of immersion in 2% osmium tetroxide in 0.1 M sodium cacodylate buffer and 0.1 M imidazole (pH 7.5) with microwave stimulation. Whole samples were next stained in 8% uranyl acetate followed by microwave stimulation and dehydration in an ethanol series of increasing concentration and transferred to an acetone solution with microwave stimulation. Samples were embedded in EMBED resin (Embed-812 resin kit, Electron Microscopy Services) and blocks allowed to polymerise at 65°C for at least 48h. Ultrathin sectioning was performed using a Reichert–Jung Ultracut Microtome, followed by transfer to copper EM grids (200 Mesh Grids, Agar Scientific). Sections were then stained with saturated uranyl acetate and Sato’s lead stain and imaged on a Phillips CM120 Biotwin transmission electron microscope or a Jeol JEM-1400 Plus electron microscope.

### Biozzi-mouse EAE

All the procedures were performed in compliance with national and institutional guidelines (UK Animals Scientific Procedures Act 1986 and the University of Cambridge, QMUL and Edinburgh Animal Care Committees). Induction of Biozzi-EAE was performed as previously described (22). Briefly, Biozzi-ABH mice were inoculated in the hindflanks twice, 7 days apart (day 0 and day 7), with spinal cord homogenate in complete Freund’s adjuvant. At day 29 mice were terminally anaesthetised with sodium pentobarbitone, and then perfused with phosphate buffered saline (PBS) followed by 4% paraformaldehyde in PBS, before spinal sections from C4-C6 were removed and immersed instantly into a 4% glutaraldehyde fix.

Post-fixation of spinal cords was done using 4% glutaraldehyde. Spinal cord sections from cervical level 5 were stained in 2% osmium tetroxide and dehydrated in a series of ethanol washes before transfer to propylene oxide. Finally, following embedding in resin and hardening at 60°C, semithin sections were cut and stained with toluidine blue in Borax solution before clearing in xylene and mounting in DPX solution. Imaging of sections was performed on Zeiss Axiovision microscope and Axiovision 4.8 software via a digital camera using an oil-immersion objective (63X), at 150 μm depth into the dorsal spinal cord, with a tile size of 152 × 114 μm.

## Supplementary Material

Data S1

MDAR Reproducibility Checklist

Movies S1 to S5

Supplementary Materials

## Data Availability

All data are available in the main text or the supplementary materials. Please see also “Data-and-analysis-File.xlsx.”
