# Supplementary material for: Myelin sheaths in the central nervous system can withstand damage and dynamically remodel
Source: Science. Author manuscript; Available in PMC 2026 Mar 18. (PMC7618902; doi:10.1126/science.adr4661)
Supplement: Supplementary Materials [file EMS212718-supplement-Supplementary_Materials.pdf]

Supplementary Materials

Materials and Methods (references 67-79 are included in M+M only)

Figs. S1 to S11

Tables S1 to S2

Movies S1 to S5

Data S1 to S2

Supplementary Materials for

Title: Myelin sheaths in the central nervous system can withstand damage and dynamically remodel

Authors: Donia Arafa[1,2,3,†,*], Julia van de Korput[1,2,4,†], Philipp N. Braaker[1,2,5,‡], Kieran P. Higgins[6,7,‡], Niels R. C. Meijns[8,‡], Katy L.H. Marshall-Phelps[1,2], Julia Meng[1,2], Daniel Soong[1,9], Eleonora Scalia[1,2], Kyle Lathem[1], Marcus Keatinge[1,2,10,11], Claire Richmond[1,2], Anna Klingseisen[1], Marja Main[1], Sarah A. Neely[1], David W. Hampton[1,2,10,11], Greg J. Duncan[12,13], Geert J Schenk[8], Marie Louise Groot[14], Siddharthan Chandran[1,2,10,11], Ben Emery[12], Antonio Luchicchi[8], Maarten H. P. Kole[6,7], Anna C. Williams[2,4], David A. Lyons[1,2,9,11,*].

Corresponding author: David.lyons@ed.ac.uk; darafa@tcd.ie

The PDF file includes:

Materials and Methods
Figs. S1 to S11
Tables S1 to S2

Other Supplementary Materials for this manuscript include the following:

Movies S1 to S5
Data S1 to S2

Materials and Methods

Zebrafish lines and maintenance

Zebrafish (*danio rerio*) were maintained under standard conditions at the Queen's Medical Research Institute BVS Aquatics facility, University of Edinburgh. All experiments were performed according to Home Office regulations under project licenses PP3290955, PP5258250 and 70-8436. Adult zebrafish were kept in a 14/10 hour light/dark cycle. Embryos were maintained in plastic petri-dishes (Fisher Scientific) at 28.5°C in 10 mM HEPES-buffered E3 embryo medium, at a maximum density of 50 eggs per dish, until 3-4 dpf, when they were arrayed into 24-well or 6-well plates. Animals were housed individually when repeat analyses were conducted on the same zebrafish and for optogenetic studies, however in all other experiments 3 larvae per well (in 24-well plates) or multiple larvae per plate were housed together. Larval zebrafish were analysed between 4 dpf and 15 dpf, before sexual differentiation in zebrafish. Throughout text and figures, 'Tg' refers to stable, germline-inserted zebrafish transgenic lines. The following transgenic lines were used in this study: Tg(mbp:TRPV1-tagRFPt) (*15*), Tg(mbp:EGFP-CAAX) (*67*), Tg(mbp:mCherry-NTR) (*21*), Tg(NBT:DsRED)(*68*), Tg(hspGFF62A:Gal4;UAS:membraneScarlet) referred to as Tg(Mauthner:Gal4;UAS:mem-mScarlet) (*69–71*), Tg(vglut2a:Gal4; 10xUAS:ChRimsonR-mScarlet)(*72*, *73*).

Single oligodendrocyte labelling

To label individual oligodendrocytes, fertilised eggs were co-injected with 5-10 pg pTol2-mbp:EGFP-CAAX DNA plasmid and tol2 transposase mRNA, at the 1-4 cell stage. Between 3-4 dpf, animals were sorted for fluorescence to identify isolated oligodendrocytes for live imaging.

Metronidazole induction of demyelination

Metronidazole (Sigma, M1547) was weighed out to prepare a fresh 10 mM stock in 2% DMSO or E3 medium only for every experiment. This 2x stock solution was then added to an equivalent volume of E3 embryo medium containing animal(s) in the wells of a 24/96-well plate or petri dishes, to make a final working concentration of 5 mM mtz in 1% DMSO or 1% DMSO only. Larvae were treated with mtz or vehicle, starting from 4 dpf, at 28.5 °C, except when animals were treated in petri-dishes at 13 dpf. After incubation in mtz, larvae were transferred to a fresh solution of E3 medium.

Capsaicin induction of demyelination

Stocks of csn (Sigma-Aldrich) were prepared to a concentration of 20 mM in 100% dimethylsulfoxide (DMSO) (Fisher Scientific) and stored at -80 °C in 20 µl aliquots. A fresh aliquot was used for each experiment. Larvae were treated by bath-application for 2 hours with the working concentration of 5 or 10 µM csn in DMSO or vehicle control (1% DMSO) at 28.5 °C, always at 4 dpf. At the end of this 2 hour incubation period, larvae were transferred to fresh E3 medium.

Zebrafish drug treatments

Drug treatments were performed by bath application. Pentylenetetrazole (ptz, Tocris, #2687) was stored at a stock concentration of 200 mM in H_2O at -20°C. For experiments, ptz was used at a working concentration of 3 mM for 18 hours, coinciding with application of csn in the case of the Tg(mbp:TRPV1-tagRFPt) model. Ptz was added at 8 hours post initial application of mtz for 16 hours in the case of the Tg(mbp:mCherry-NTR) model. Veratridine (Tocris, #2918) was stored at a stock concentration of 20mM in DMSO at -20°C. For experiments to enhance sodium channel activity, veratridine was used at a concentration of 2.5 µM for 24 hours coinciding with application of mtz, or at a concentration of 1 µM for 48 hours coinciding with application of mtz.

Tricaine methanesulfonate (MS222 in text and figures, Thermo Scientific, # 118000500), was prepared to a stock concentration of 15 mM (in H_2O + 2% 1 M Tris buffer pH 9) and stored at 4°C. For experiments to reduce sodium channel activity, MS222 was used at a concentration of 0.2 mM, and animals were treated for 48 hours coinciding with application of mtz, with daily medium changes in the Tg(mbp:mCherry-NTR) model. Phenytoin (ApexBio) was stored at a stock concentration of 10 mM in DMSO and used at a working concentration of 80 μM for experiments. Animals were treated for 48 hours coinciding with application of mtz in the Tg(mbp:mCherry-NTR) demyelination model.

Zebrafish live imaging

Animals were anaesthetised in 0.6 mM MS222 in E3 embryo medium for live imaging. For single timepoint analyses, larvae were positioned laterally on glass coverslips using a forceps, immobilised in 1.3-1.5% low melting-point agarose. Coverslips were mounted over a slide using high-vacuum silicone grease to create a well containing E3 embryo medium and 0.6 mM MS222. Z- stacks were obtained using a Zeiss LSM880 Airyscan confocal microscope using FAST mode, and a 20X or 40X objective (Zeiss Plan-Apochromat 20X dry, numerical aperture = 0.8; Zeiss C-Apochromat 40X water, numerical aperture = 1.2).

All images of myelin, using Tg(mbp:EGFP-CAAX), following drug treatments were taken from a lateral view of the spinal cord, by lining up the bottom of the field of view at the level of the urogenital opening, unless otherwise stated. To repeat image individual oligodendrocytes, larvae were removed from the agarose following image acquisition and returned to E3 embryo medium and checked for signs of impaired health or abnormal swim behaviour. Between imaging sessions zebrafish were returned to the 28.5°C incubator. The exact somite position was noted in the first imaging session and used to orient the imaging window for the subsequent imaging

sessions. Other landmarks within the same channel (such as neighbouring cells) or another channel (such as the Mauthner axon/oligodendrocyte cell bodies) were then used as additional references from the previous images to find the exact same cell/location for the following timepoints. This was again double-checked at the analysis stage by cross-referencing images taken from different timepoints to ensure the same cell/region had been captured over time.

For longer term imaging in timelapse experiments, animals were immobilised using the neuromuscular blocker α-bungarotoxin (Tocris) at 4 dpf. α-bungarotoxin was prepared to a stock concentration of 1 mg/mL, and 20 µl aliquots stored at -20°C. For experiments, all embryo medium was first removed from animals in a glass dish before bath application with α-bungarotoxin for 1 minute each and re-immersion in E3 embryo medium. For overnight imaging, immobilised animals were maintained in 1.5% low melting-point agarose in a plastic 50 mm dish containing E3 embryo media. The incubator was set to 28°C. Z- stacks were obtained using FAST mode, and a 20X objective (Zeiss Plan-Apochromat 20X water-dipping, numerical aperture = 1.0), and images acquired every 20 minutes for up to 16 hours. Where imaging was conducted during csn treatment, csn was added to the petri dish by bath application and removed between imaging timepoints. In some cases, animals were then removed from the microscope and re-imaged up to 35 hours post-treatment with csn, ensuring that animal health was still optimal.

Optomotor-based stimulation of swimming in zebrafish

Tg(mbp:mCherry-NTR); Tg(mbp:EGFP-CAAX) zebrafish were sorted for NTR (mCherry) and GFP expression at 4 dpf and transferred to petri-dishes containing 5 mM mtz. To induce swimming using the OMR, zebrafish were presented with a pattern of black and white bars, 2mm wide moving at a speed of 20mm per second, which stimulates swimming in the direction of the moving bars (**Movie S2**). Persistent swimming was maintained by alternating the direction of the

moving bars at 20 second intervals. Petri-dishes containing animals where the moving bars were obscured from view were used as controls. Zebrafish were maintained in these conditions for 24 hours before being anaesthetised for live imaging of myelin. The moving pattern was configured using custom software available here https://github.com/klathem/Optical-Treadmill.

Optogenetic-based stimulation of glutamatergic neurons and assessment of myelin swellings

Optogenetic-based stimulation was conducted similarly to previous studies (*35*). In brief, Tg(mbp:mCherry-NTR; mbp:EGFP-CAAX; Tg(vglut2a:Gal4; 10XUAS:ChRimsonR-mScarlet) (*72*, *73*) zebrafish were screened by fluorescence for expression of NTR (mCherry) and membrane-tethered GFP in oligodendrocytes, and the channel rhodopsin ChR (mScarlet) in neurons. Zebrafish were subsequently arrayed one animal per well into a 96-well plate already containing 5 mM mtz at 4 dpf. Controls (siblings) were animals in the same plate that were opto-stimulated but did not express ChR. To test response to opto-stimulation, swim behaviour was assessed in ChR-expressing animals following light-exposure and those animals exhibiting a reliable response were used for the experiment. All zebrafish in the plate were exposed to light by widefield illumination using a 569 nm LED every 15 minutes for 24 hours with a motorised Zeiss Observer T1 system, as previously described (*35*). The plate was sealed to prevent evaporation, and zebrafish were maintained at 28.5 °C for the duration of the stimulation. At the end of the long-term stimulation, zebrafish were then anaesthetised and imaged by confocal microscopy on an LSM880 using a 20X objective.

Zebrafish image analysis

Most image processing and analysis was conducted using Fiji (Version 2.3.0 /1.53f). Z-stacks were converted to maximum intensity projections (MIPs), unless otherwise stated. Images were then rotated so that the anterior is always displayed on the left and dorsal is at the top of the

image. For display purposes, brightness and contrast have been adjusted in figures, but this was unchanged between conditions prior to any analysis. Prior to any manual analyses images were randomised and the analyser blinded to experimental condition.

Sheath length was measured manually using the segmented line tool in Fiji. Comparison of individual sheaths over time was done by carefully comparing images from different timepoints post-measurements to ensure that the same cell/sheath was being compared. For dorsal oligodendrocytes, only a subset of sheaths from each cell that were isolated and visible at all timepoints were included in analyses. To quantify myelin damage in entire ventral spinal cords following drug treatments in the Tg(mbp:mCherry-NTR) model, the number of myelin swellings that protruded above the ventral spinal cord in a maximum intensity projection spanning a 235 µm section of spinal cord were counted and displayed graphically per 100 µm.

To assess myelin disorder, automated segmentation was performed using a custom pipeline designed using Arivis Vision 4D. Maximum intensity projections of z-stacks of the mbp:EGFP-CAAX reporter were first imported into the software and used to train a machine-learning based algorithm. The overall signal within each image was detected ('myelin'). Next, object classes were manually defined and categorised into linear, normal-appearing myelin running along the spinal cord (termed 'ordered' throughout this study, predominantly composed of regions with more horizontally aligned pixels) and regions of myelin swelling (termed 'disordered' throughout this study, predominantly including more vertically aligned pixels). Examples of segmentation are in **Fig. S5**. The batch analysis module was used to process the images using the pipeline. Data were expressed as the ratio between the amount of disordered:ordered plus disordered pixels detected in the 'myelin' compartment and this value per image or animal as specified in figure legends was used for statistics.

To assess % myelination/demyelination of the Mauthner axon from images taken of Tg(mbp:EGFP-CAAX) animals, MIPs were cropped to only include the top edge of the myelin surrounding Mauthner axon (height of 6 μm for each image). The 'axon trace'(*71*) tool was used to trace the highest intensity profile along the image and the 'plot histogram' function in Fiji with a grey value threshold of 2000 used to then determine % myelination along this trace.

To assess myelin swellings following optogenetic stimulation, z-stacks containing the dorsal half of the spinal cord closest to the objective were scrolled through to manually count individual myelin swellings. Following counting swellings, the red channel of the image was used to exclude any counted that were oligodendrocyte cell bodies. For analyses of ventral spinal cord, swellings that were visible above the anatomically identifiable Mauthner axon were manually counted on maximum intensity projections. All counts were performed using Fiji.

To assess swelling burden in single oligodendrocytes, number of swellings and unaffected sheaths were manually quantified through the z-stacks using cell counter plugin on Fiji. The area of swelling was assessed by manually tracing swellings based on the stack in which they looked largest. The average swelling size is the average area of all swellings per cell, while the total swelling area is the sum of the area of all swellings per cell. We always assessed one cell per animal, unless otherwise stated in the figure legend.

Zebrafish live imaging statistics and reproducibility

After sorting for fluorescence, for all experiments larvae were randomly assigned to different treatment conditions. During imaging, animals from independent groups were imaged in an alternating pattern to preclude any effects of time on the parameters being assessed. Manual analyses were always conducted blinded to treatment condition where the effects of drug candidates were being assessed in Tg(mbp:EGFP-CAAX) animals, by two independent blinded experimenters. No data points were excluded from analysis due to variability. All data presented

in this study are from experiments conducted over multiple clutches (2-8) of zebrafish over multiple days.

Sample sizes are comparable to what has previously been used for zebrafish live imaging studies (*15*). Unless otherwise stated in the figure legend, N refers to an individual animal, and if multiple cells were analysed per animal values for these were averaged and noted in figure legends. Comparisons of datasets were conducted by parametric statistical analyses unless otherwise stated, using GraphPad Prism GraphPad Software, Inc., San Diego, United States. In graphs, normally distributed data are displayed as mean ± 95% confidence interval (CI) unless otherwise stated. A difference was considered statistically significant when $P < 0.05$.

Cortical organotypic slice culture preparation and culturing

To prepare slice cultures we used male and female C57BL/6JRj mice. All animal procedures were performed with the approval from the Royal Netherlands Academy of Arts and Sciences (KNAW) Animal Ethics Committee (DEC) and Central Authority for Scientific Procedures on Animals (CCD, license AVD80100202216329), and overseen by the Animal Welfare Body (IvD, NIN.22.21.02). Cortical organotypic slice cultures were prepared from 4-5 day old mouse pups by anaesthetizing animals via hypothermia followed by decapitation with scissors. The brain was extracted and placed in ice-cold dissection solution consisting of 98% GBSS (in mM) 137 NaCl, 1.5 $CaCl_2$, 0.2 KH_2PO_4, 0.3 $MgSO_4$, 2.7 $NaHCO_3$, 5 KCl, 1 $MgCl_2$, 0.85 Na_2HPO_4, 5.6 D-glucose), 1% (0.1 M stock) kynurenic acid, and 1% (2.5 M stock glucose), sterile filtered with 0.2 µm filtration flasks, and adjusted to pH 7.2 and 320 mOsm. Under a dissection microscope, brains were cut down the midline with a scalpel and then sectioned via McIlwain Tissue Chopper to obtain 300 µm thick coronal slices. Slices were quickly transferred to hydrophilic PTFE membrane inserts (Merck-Millipore, PICMORG50) in 6-well plates and

cultured at 35 °C and 5% CO2 in culturing medium consisting of 47.75% MEM (Thermo Fisher Scientific # 11575032), 25% HBSS (Thermo Fisher Scientific # 24020133), 25% heat-inactivated horse serum (Thermo Fisher Scientific # 26050088), 1% (2.5 M stock) D-glucose, and 1.25% (1 M stock) HEPES (Sigma-Aldrich H3375), sterile filtered, and adjusted to pH 7.2 and 320 mOsm. Medium was changed 3 x a week with fresh equilibrated medium. AAV-MBP:mem-Tdtomato was applied directly to slices cultures at 7 DIV to selectively label oligodendrocytes and allow for visualization of the membrane structure of myelin sheaths with the myelin basic protein (MBP)-specific membrane-bound tdTomato fluorophore (*42*).

Cortical organotypic slice culture live two-photon imaging

14-21 days in vitro (DIV) cultures were placed in the recording chamber of a two-photon microscope (Femto-3D-RD, Femtonics Inc., Budapest, Hungary), perfused with carbogen-bubbled recording solution (in mM: 125 NaCl, 25 $NaHCO_3$, 1.25 NaH_2PO_4, 3 KCl, 25 D-Glucose, 2 $CaCl_2$, 1 $MgCl_2$). TdTomato labelled oligodendrocytes were visualized via a Ti:Sapphire laser (Chameleon Ultra II; Coherent, Inc.) tuned to 1030 nm, cells were imaged using a 1.0 NA 20x lens (Olympus) with a voxel size of 0.3 x 0.3 x 1 µm (X/Y/Z) at 15 min intervals. Image acquisition took place in MES software (Femtonics Inc., version 6.3.7902). Acquired time-lapse images were imported into Fiji (Fiji 64 bit; ImageJ version 1.54p; RRID: SCR_002285) and swellings were manually counted. The SNT plug-in was used to measure internode length (*74*). All slice culture data presented in this study were conducted on 3 slices per condition (biological replicates), derived from 2-3 animals across separate culturing preparations. N refers to individual slices and *n* refers to single oligodendrocytes (technical replicates) unless otherwise stated. All statistical comparisons were made using GraphPad Prism and all data in graphs are displayed as ± 95% CI unless otherwise stated.

LPC induction of demyelination

LPC (Sigma #L4129) was prepared to a concentration of 10 mg/ml in a carbogen bubbled recording solution and loaded into a glass patch pipette. Brief (several seconds) positive pressure was applied to the pipette to locally perfuse the region of imaging with LPC. The final bath concentration will be a magnitude of order lower once LPC has diffused into solution (~1.0 mg/ml). Control imaging sessions used pressure applications with pipettes containing recording solution without LPC.

TTX treatment in cortical organotypic slice cultures

TTX (Bio-Techne #1069) was dissolved in water to a final stock concentration of 5 mM and stored at –20 °C until use. Fresh aliquots were used to make a working concentration of carbogen bubbled recording solution containing 1 µM TTX for each experiment. Organotypic slice cultures were treated with 1 µM TTX for 10 min prior to induction of demyelination via LPC and through the duration of imaging.

Immunohistochemistry and confocal imaging of cortical organotypic slice cultures

Cortical organotypic slice cultures were removed from the imaging chamber following imaging sessions and fixed with 4% PFA in PBS for 20 min followed by 3 x 10 min washes in PBS. Slices were then blocked for 2 hr in PBS containing 0.5% Triton X-100 and 10% normal goat serum. Next, slices were incubated 0.25% Triton X-100 and 5% normal goat serum in PBS containing primary antibodies, overnight at room temperature while shaking. Primary antibodies used: Guinea pig polyclonal antibody to RFP (Synaptic Systems, Cat: 390 004, RRID: AB_2737052, 1:500), Mouse monoclonal antibody to pan-axonal neurofilament marker SMI-312 (Eurogentec Cat: SMI 312P 050, 1:6000), Chicken polyclonal IgY antibody to MBP (Aves Labs , Cat: MBP, RRID: AB_2313550, 1:200). The following day, slices washed 3 x 10 min in PBS.

After washing, slices were incubated in PBS containing secondary antibodies for 2 h at room temperature while shaking and protected from light. Slices were finally washed in PBS 3 x 10 min before mounting with Vectashield mounting medium containing DAPI (Vector Laboratories #H-200). Secondary antibodies used: goat anti-mouse IgG, IgM (H+L) secondary antibody, Alexa Fluor 488 (A10684, RRID: AB_2534064). goat anti-guinea pig IgG (H+L) highly cross-adsorbed secondary antibody, Alexa Fluor 594 (A11076, RRID: AB_141930). Goat anti-chicken IgY (H+L) secondary antibody, Alexa Fluor 647 (A21449, RRID: AB_2535866).

Stained slices were imaged on a Leica SP8 confocal microscope using a 40x 1.3 NA oil-immersion lens and running LASX (3.5.7). Images were acquired using sequential scans of individual channels using step sizes of 0.299 μm along the z-axis and at a 2048 x 2048 pixel resolution. Images were imported in Fiji and maximum projection function was used to generated for use in Figure 5.

Preparation of human tissue slices from MS donors

For live imaging experiments we used acute slices from post-mortem donors, acquired from the Netherlands Brain Bank (www.brainbank.nl, 2009/148). The whole study was performed in strict compliance with ethical requirements of the Amsterdam University medical centers/VU medical centrum, Amsterdam and the Netherlands Code of Conduct for Research Integrity and the Declaration of Helsinki. Informed consent was asked to the donors. For tissue preparation, we sectioned corpus callosum brain tissue (selecting an area between the genu and the trunk of the corpus callosum) from 3 MS donors with short post-mortem delay (**Table S2**), using a protocol previously published (*43*). In short, immediately after resection samples were cooled for ten minutes in ice-cold N-methyl-D-glucamine (NMDG) buffer comprising: 93 mM NMDG, 2.5 mM KCl, 1.2 mM NaH_2PO_4, 20 mM HEPES, 12 mM N-acetyl-L-cysteine (NAC), 5 mM

sodium ascorbate, 3 mM sodium pyruvate, 10 mM $MgSO_4$, 30 mM $NaHCO_3$, and 25 mM glucose; pH 7.4 before being cut to create flat tissue surfaces. Protective slice recovery was performed in oxygenated NMDG at room temperature for three minutes with carbogen (95% O2/5% CO_2, at a 0.1 L/min flow rate), followed by a 60-minute oxygenated incubation at room temperature in 50 mL of HEPES holding solution, containing: 92 mM NaCl, 2.5 mM KCl, 1.2 mM NaH_2PO_4, 20 mM HEPES, 1 mM NAC, 5 mM sodium ascorbate, 3 mM sodium pyruvate, 0.5 mM $MgSO_4$, 1 mM $CaCl_2$, 30 mM $NaHCO_3$, and 25 mM glucose; pH adjusted to 7.4. Subsequently, each slice was placed on a #1.5H glass coverslip (µ-Dish 35 mm, high glass bottom, ibidi, Gräfelfing, Germany) for live-cell recording, secured by a custom-cut sponge or harp to reduce tissue drift during imaging.

Third harmonic generation microscopy

To image the samples using THG microscopy, sample holders with prepared brain slices were transferred to a stage-top incubation chamber (H301-PRIOR-H117, OKOLAB S.R.L.), maintained at a constant temperature of 37 °C under continuous carbogen-95% O_2/5% CO_2 flow). For live-cell imaging, continuous perfusion with artificial cerebrospinal fluid (aCSF) was conducted at a rate of 1 mL/min using peristaltic pumps (ISM832C, ISMATEC, Cole-Parmer GmbH). The aCSF was comprised of 125 mM NaCl, 15 mM KCl, 2 mM CaCl, 1.25 mM NaH_2PO_4, 1 mM $MgSO_4$, 26 mM $NaHCO_3$, 10 mM $C_6H_{12}O_6$; with a pH maintained at 7.4. Time-lapse recordings were conducted with a 200 × 200 µm or 400 × 400 µm FOV within a 1000 × 1000-pixel image at an acquisition speed of one frame per 1.8 seconds. THG images were generated every 5 minutes for a total of 1.5~2 hours. Recordings, including THG time-lapsed z-stacks, were initially saved as 8-bit grayscale BMP files and THG channels were automatically processed for histogram normalization before being converted into TIFF stacks using the Fiji software suite for further data analysis.

Swelling imaging and analysis of human tissue slices

Images were imported in Fiji and rotated to have axons oriented horizontally and corrected for XY drift. Swellings with a detectable outline were included for tracking longitudinally for 1.5 hours. A custom-made ImageJ macro was used to correct potential drift in Z throughout the time-series. Manually, outlines around the myelin swellings were drawn with the Fiji polygon function to measure swelling areas. The area of swelling was assessed by manually tracing swellings based on the stack in which they looked largest. LOESS smoothing was applied to spaghetti plots.

Human post-mortem brain tissue histology

Post-mortem brain tissue from MS patients and non-neurological controls were provided by a UK prospective donor scheme with full ethical approval from the UK Multiple Sclerosis Society Tissue Bank (MREC/02/2/39) and from the MRC-Edinburgh Brain Bank (16/ES/0084). MS diagnosis was confirmed by neuropathological means by F. Roncaroli (Imperial College London) and Prof. Colin Smith (Centre for Clinical Brain Sciences, Centre for Comparative Pathology, Edinburgh) with no signs of confounding neurodegenerative diseases and clinical history was provided by R. Nicholas (Imperial College London) and Prof. Colin Smith. **Table S1** includes anonymised details on samples used.

For histological analysis, 4 μm sequential sections from paraffin tissue blocks of 2 cm × 2 cm × 1 cm were stored at room temperature. All brain donor samples were from cortical hemisphere white matter. The MS samples chosen had confirmed white matter demyelinated lesions from primary motor, frontal, or periventricular white matter regions and non-MS samples were from primary motor and frontal white matter. The MS samples chosen had confirmed white matter

demyelinated lesions. Overall, 7 male and 6 female samples were used (controls: 4 male and 2 female samples, MS: 3 male and 4 female samples).

Human post-mortem brain tissue immunofluorescent staining

Paraffin sections were rehydrated and microwaved for 15 min in Vector Unmasking Solution for antigen retrieval (H-3300, Vector). For immunofluorescence, sections were incubated with Autofluorescent Eliminator Reagent (2160, MERCK-Millipore) for 1 min and briefly washed in 3% hydrogen peroxide after antigen retrieval, then washed and blocked for 1 hour with 10% normal horse serum, 0.5% Triton-X in TBS. Primary antibodies were diluted in serum block and incubated overnight at 4 °C in a humidified chamber. Primary antibodies used: rabbit recombinant monoclonal IgG antibody to PLP (Clone EPR23504-106, AB254363, Abcam, 1:100) and chicken polyclonal IgY antibody to NF-H (Clone Poly28226, Biolegend, Cat: 822601, RRID: AB_2564859, 1:100). The next day, sections were incubated with Alexa Fluor secondary antibodies (Thermo Fisher Scientific, 1:1000) for 1 hour at room temperature and counterstained with Hoechst or DAPI for nuclear visualisation. Secondary antibodies used: goat anti-chicken IgY secondary antibody, Alexa Fluor 647 (A21449, RRID: AB_2535866), donkey anti-rabbit IgG highly cross-absorbed secondary antibody, Alexa Fluor 568 (A10042, RRID: AB_2534017), donkey anti-chicken IgY (H+L) highly cross-absorbed secondary antibody, Alexa Fluor 488 conjugate, A78948, RRID: AB_2921070. All slides were mounted using SouthernBiotech Fluormount-G slide mounting medium (Cambridge Bioscience, #0100-01).

Human post-mortem brain tissue imaging

Entire sections were imaged on a Zeiss Axioscan Slidescanner with a 40x objective (Zeiss Plan-Apochromat 40x, numerical aperture = 0.95) and lesions were identified by a lack of PLP stain using Zeiss ZEN lite imaging software. High-resolution images were obtained using an LSM880

confocal microscope using a 63x objective (Zeiss Plan-Apochromat 63x oil, numerical aperture = 1.4). Z-stacks were acquired with an optimal z-step according to the experiment, all exemplar images are maximum intensity projections.

Human post-mortem brain tissue quantification

Analysis was done using QuPath version 0.4.4 and Fiji ImageJ 64-bit version 2.14.0/1.54f software. Lesion borders were drawn on QuPath based on distinct areas of demyelination as seen by the PLP stain. The perilesional area was created using a 200 µm border from the lesion edge, and within this area we randomly selected 5 locations with longitudinally tracking axons (to ensure swellings could be identified) and created 150 µm x 150µm boxes to count the number of swellings, see **Fig. S11**. For NAWM and control non-MS tissue, we randomly selected 5 apparently normally myelinated locations by PLP staining. Demyelinated lesion types were defined based on standard pathology classification into active, chronic active, and chronic inactive lesion types (*75*) using number and location of infiltrating immune cells with sharpness of the demyelinated lesion edge. Following quantification of 5 locations, we summed the total number of myelin swellings per lesion type/NAWM/control tissue (112,500 μm^2). Of note, the 4 µm thick sections do not allow reconstruction of whole oligodendrocyte morphology, so data is displayed as number of swellings per area.

Genetic ablation of Myrf and tissue processing

Mice were maintained in the Oregon Health & Science University animal facility in pathogen-free conditions on a 12-hour light/dark cycle. Myrf floxed mice (*76*) on a C57BL/6 background were crossed to the Plp1-CreERT mouse line (*77*) (Jax line 005975) to generate $Myrf^{Fl/Fl}$; Plp1-$CreERT^{+}$ ($Myrf^{\Delta iPLP1}$) mice and $Myrf^{Fl/Fl}$; $CreERT^{-}$ control littermates. At eight weeks of age all mice were treated with tamoxifen (T5648, Sigma, 100mg/kg i.p. for five consecutive days) to

induce recombination of the floxed *Myrf* allele and subsequent demyelination in $CreERT^+$ animals. At eight weeks post tamoxifen mice were deeply anesthetized with ketamine (400 mg/kg) and xylazine (60 mg/kg) and transcardially perfused with 10 mL of phosphate buffered saline (PBS) followed by 40mL of freshly prepared 4% paraformaldehyde in PBS. All animal procedures were performed in accordance with, and approved by, the Institutional Animal Care and Use Committee of OHSU.

Tissue was processed for electron microscopy as previously described (*20*, *78*). Dissected optic nerves were postfixed overnight in 2% paraformaldehyde and 2% glutaraldehyde in PBS before being stored for one to two weeks in 1.5% paraformaldehyde, 1.5% glutaraldehyde, 50mM sucrose, 22.5mM $CaCl_2$ in 0.1M cacodylate buffer. Nerves were infiltrated with 2% osmium tetroxide (19190, Electron Microscopy Sciences) and 1.5% potassium ferrocyanide (25154-20, Electron Microscopy Sciences) using a Biowave Pro+ microwave (Ted Pella), stained with 0.5% uranyl acetate (22400, Electron Microscopy Sciences), dehydrated in successive grades of acetone and embedded in EMbed 812 (14120, Electron Microscopy Sciences). 60nm sections two millimetres from the optic chiasm were mounted on copper grids (T400-Cu, Electron Microscopy Sciences) and counter stained with 5% Uranyl Acetate followed by Reynold's Lead Citrate (80 mM $Pb(NO_3)_2$ 17900-25, Electron Microscopy Sciences) and 120mM Sodium Citrate (21140, Electron Microscopy Sciences). Grids were imaged at 4800x on a FEI Tecnai T12 transmission electron microscope with a 16 Mpx camera (Advanced Microscopy Techniques Corp). Quantification of large vacuoles/swellings following *Myrf* ablation was done manually on sections prepared from $Myrf^{\Delta iPlp1}$ (average area analysed per animal =28372 um^2) and $Myrf^{fl/fl}$ animals (average area analysed per animal =23605 um^2) 10 weeks post-tamoxifen.

Zebrafish electron microscopy

Zebrafish samples were prepared for electron microscopy as previously described(*15*, *21*, *79*). Briefly, primary fixation of terminally anaesthetised zebrafish was performed by immersion in 4% paraformaldehyde (Agar Scientific, #R1026, EM grade) + 2% glutaraldehyde (Agar Scientific, #R1020, EM grade) in 0.1 M sodium cacodylate buffer (pH 7.4), with microwave stimulation. Following overnight incubation, secondary fixation consisted of immersion in 2% osmium tetroxide in 0.1 M sodium cacodylate buffer and 0.1 M imidazole (pH 7.5) with microwave stimulation. Whole samples were next stained in 8% uranyl acetate followed by microwave stimulation and dehydration in an ethanol series of increasing concentration and transferred to an acetone solution with microwave stimulation. Samples were embedded in EMBED resin (Embed-812 resin kit, Electron Microscopy Services) and blocks allowed to polymerise at 65°C for at least 48h. Ultrathin sectioning was performed using a Reichert–Jung Ultracut Microtome, followed by transfer to copper EM grids (200 Mesh Grids, Agar Scientific). Sections were then stained with saturated uranyl acetate and Sato's lead stain and imaged on a Phillips CM120 Biotwin transmission electron microscope or a Jeol JEM-1400 Plus electron microscope.

Biozzi-mouse EAE

All the procedures were performed in compliance with national and institutional guidelines (UK Animals Scientific Procedures Act 1986 and the University of Cambridge, QMUL and Edinburgh Animal Care Committees). Induction of Biozzi-EAE was performed as previously described (*22*). Briefly, Biozzi-ABH mice were inoculated in the hindflanks twice, 7 days apart (day 0 and day 7), with spinal cord homogenate in complete Freund's adjuvant. At day 29 mice were terminally anaesthetised with sodium pentobarbitone, and then perfused with phosphate

buffered saline (PBS) followed by 4% paraformaldehyde in PBS, before spinal sections from C4-C6 were removed and immersed instantly into a 4% glutaraldehyde fix.

Post-fixation of spinal cords was done using 4% glutaraldehyde. Spinal cord sections from cervical level 5 were stained in 2% osmium tetroxide and dehydrated in a series of ethanol washes before transfer to propylene oxide. Finally, following embedding in resin and hardening at 60°C, semithin sections were cut and stained with toluidine blue in Borax solution before clearing in xylene and mounting in DPX solution. Imaging of sections was performed on Zeiss Axiovision microscope and Axiovision 4.8 software via a digital camera using an oil-immersion objective (63X), at 150 μm depth into the dorsal spinal cord, with a tile size of 152 x 114 μm.

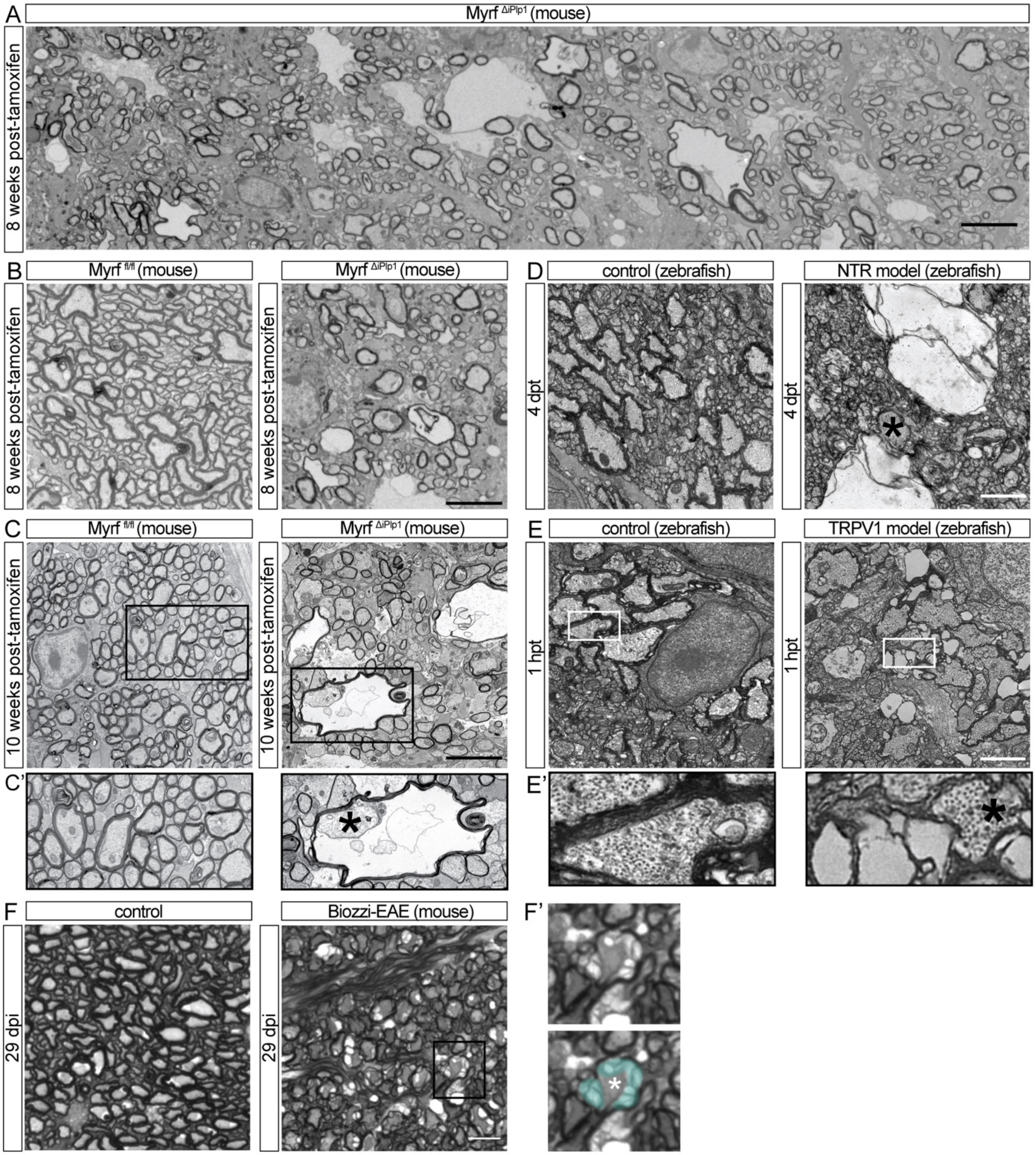

Fig. S1. Myelin swelling occurs in rodent and zebrafish models of demyelination

(A) Transmission electron microscopy overview of Myrf$^{\Delta iPlp1}$ optic nerve 8 weeks post-treatment with tamoxifen. Scale bar = 5 µm.

(B) Transmission electron microscopy images of Myrf$^{fl/fl}$ and Myrf$^{\Delta iPlp1}$ optic nerves 8 weeks post-treatment with tamoxifen. Scale bar = 5 µm.

(C) Transmission electron microscopy images of Myrf$^{fl/fl}$ and Myrf$^{\Delta iPlp1}$ optic nerves 10 weeks post-treatment with tamoxifen. Scale bar = 5 µm. C’ = cropped image from C where asterisk = axon.

(D) Transmission electron microscopy images of DMSO- and mtz-treated Tg(mbp:mCherry-NTR) zebrafish at 4 days post-treatment (dpt) showing swelling myelin in close association with axons. Asterisk = axon. Scale bar = 1 µm. dpt = days post-treatment.

(E) Transmission electron microscopy images of DMSO and csn-treated Tg(mbp:TRPV1-tagRFPt) zebrafish at 1 hour post-treatment (hpt) showing swelling myelin. Scale bar = 1 µm. E’ = cropped image from E where asterisk = axon. hpt = hours post-treatment.

(F) Images of toluidine blue-stained semithin sections of control mouse dorsal spinal cord and following EAE-induction at 29 days post induction (dpi) showing swelling myelin in close association with axons. Scale bar = 10 µm. F’ = cropped image from F where swellings are pseudocoloured and asterisk = axon.

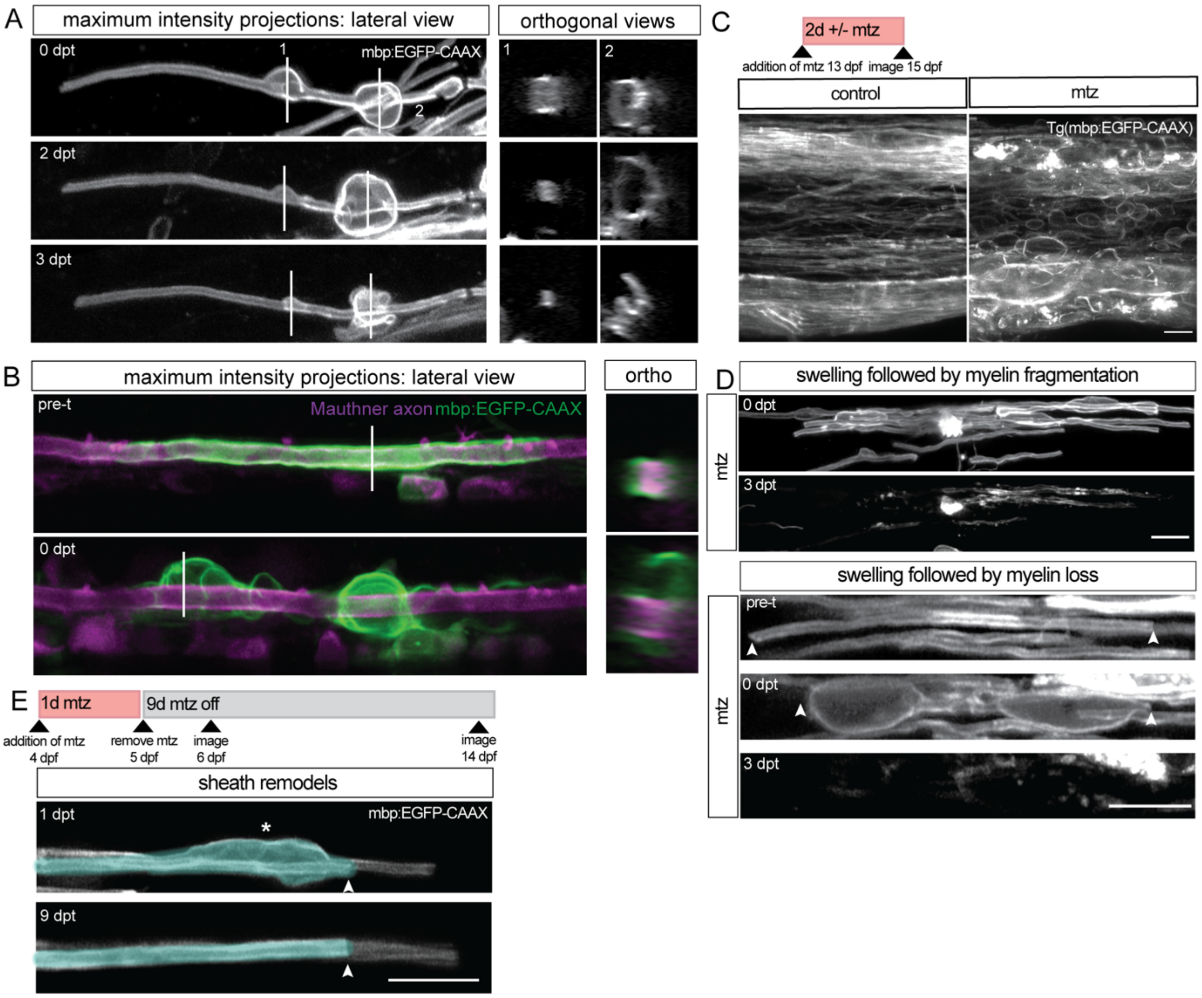

Fig. S2. Myelin swelling in Tg(mbp:mCherry-NTR) zebrafish by live imaging in longitudinal and optical cross-section

(A) Confocal images of individual myelin sheaths from an animal treated for 1 day with mtz, imaged over time where the sheath persists, showing an example of a swelling that reduces over a 4 day period (left) and an example of a swelling that increases in size between 1-3 days after the start of mtz treatment (right). Orthogonal views are provided to illustrate the change in size over time, where vertical lines indicate where views were taken.

(B) Confocal images of myelin (mbp:EGFP-CAAX) and the Mauthner axon (Tg(Mauthner:Gal4;UAS:mem-mScarlet)) in the same animal pre-treatment and post 2 days of treatment with 5 mM mtz. Orthogonal views are provided to illustrate the change in size over time, where vertical lines indicate where the orthogonal views were taken.

(C) Schematic of experimental timings for imaging myelin swelling induced at 13 days post fertilization (dpf) in the zebrafish Tg(mbp:mCherry-NTR) model. Confocal images myelin in the spinal cord labelled by the transgenic reporter Tg(mbp:EGFP-CAAX) in Tg(mbp:mCherry-NTR)-expressing animals treated with vehicle or mtz for 2 days at 13 dpf. Asterisks = swellings. Scale bar = 10 μm.

(D) Confocal images of individual oligodendrocytes from animals treated for 1-2 days with 5 mM mtz, imaged over time, showing an example of sheath fragmentation and loss. White arrows indicate ends of the myelin sheath. Scale bar = 10 μm. dpt = days post-treatment.

(E) Schematic of experimental timings for imaging longer-term recovery of myelin swellings in individual oligodendrocytes in the zebrafish Tg(mbp:mCherry-NTR) model.Confocal images of the same individual myelin sheaths from animals treated for 1 day with 5 mM mtz, imaged over time up to 9 days post-treatment where the swelling recovers. Asterisks indicate location of swellings. White arrows indicate ends of the myelin sheath. Pseudo-colouring added to clarify the location of the myelin sheath. Scale bar = 10 μm. dpt = days post-treatment.

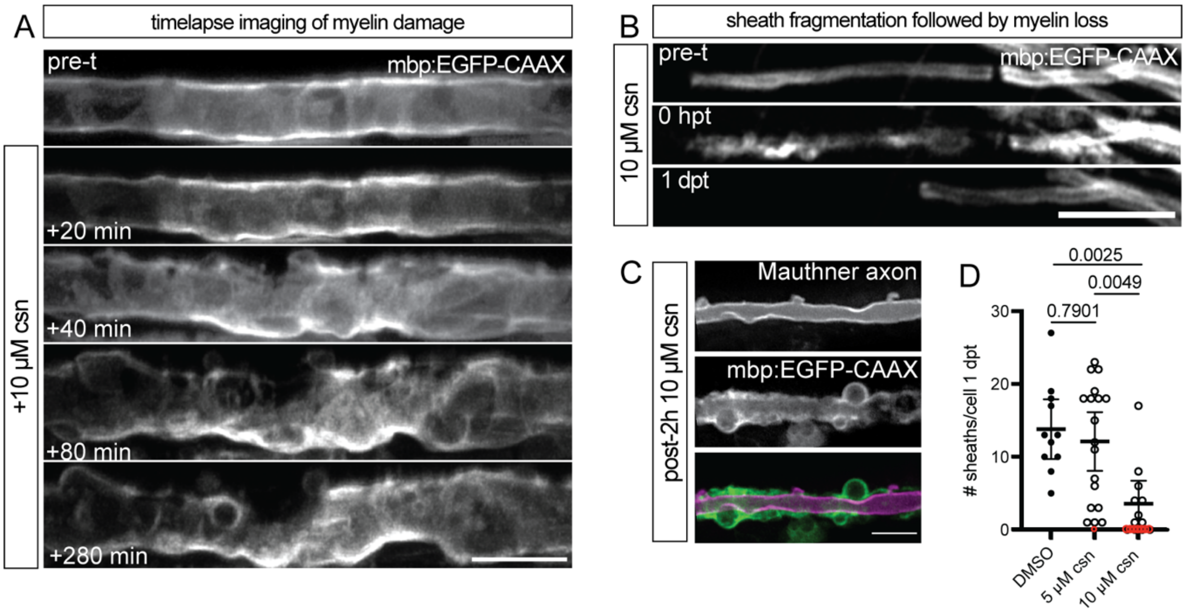

Fig. S3. Damage to individual myelin sheaths in the Tg(mbp:TRPV1-tagRFPt) model

(A) Timelapse series of confocal images of mosaically-labelled oligodendrocytes and their myelin from pre-treatment (4 dpf) with 10 µM csn until 280 minutes later. Scale bar = 10 µm.

(B) Confocal images of myelin sheaths belonging to dorsal oligodendrocytes, from animals treated for 2 hour with 10 µM csn imaged over time pre-treatment, immediately post-treatment, and 1 day post treatment (dpt), showing rapid loss of myelin sheaths. Scale bars = 10 µm.

(C) Confocal images of myelin (mbp:EGFP-CAAX) and the Mauthner axon (Tg(Mauthner:Gal4;UAS:mem-mScarlet)) immediately post 2 hour treatment with 10 µM csn. Scale bar = 10 µm.

(D) Quantification of dorsal oligodendrocyte sheath number in Tg(mbp:TRPV1-tagRFPt) animals treated with 1% DMSO, 5 µM csn or 10 µM csn when cells were imaged 1 dpt. One-Way ANOVA with Tukey's multiple comparisons test. n = 11 DMSO-treated animals, n = 18 cells from 16 5 µM csn-treated animals, n = 12 cells from 11 10 µM csn-treated animals. Red datapoints indicate cells that died following treatment, which were not included for statistical assessment of changes in sheath number per cell. Error bars are mean ± 95% CI.

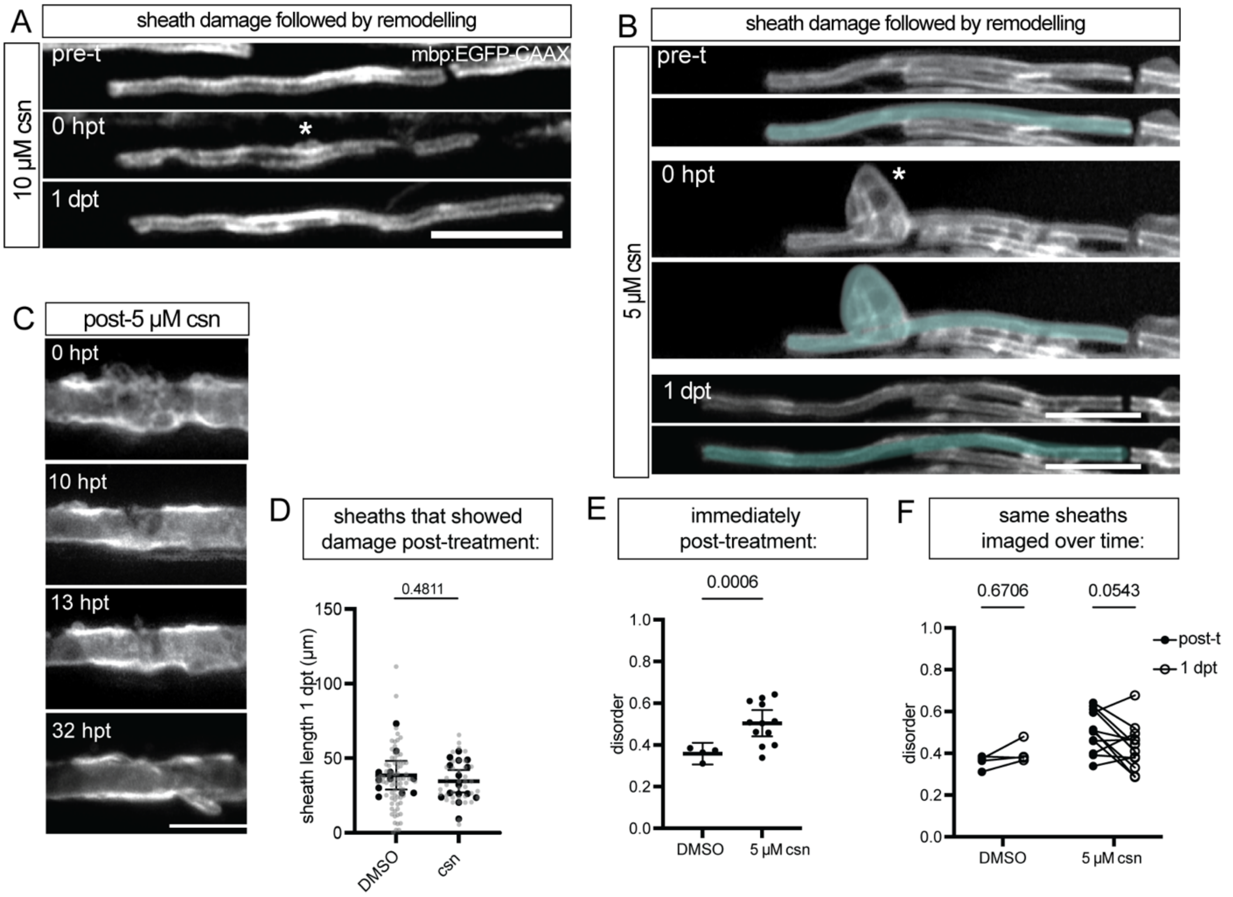

Fig. S4. Individual myelin sheaths can persist and remodel following damage in the Tg(mbp:TRPV1-tagRFPt) model

(A) Confocal images of myelin sheaths from a dorsal oligodendrocyte, from an animal treated for 2 hours with 10 µM csn imaged over time; Pre-treatment, immediately post-treatment, and 1 day post treatment (dpt), showing myelin sheaths that persist through damage and remodel. Scale bars = 10 µm.

(B) Confocal images of myelin sheaths belonging to dorsal oligodendrocytes, from animals treated for 2 hours with 5 µM csn imaged over time; pre-treatment, immediately post-treatment, and 1 dpt. Asterisks = regions of damage. Pseudocolouring added to aid with following individual myelin sheaths over time. Scale bars = 10 µm.

(C) Timelapse series of confocal images of mosaically-labelled Mauthner-myelinating oligodendrocytes and myelin following treatment with 5 µM csn until 24 hours post-treatment, re-imaged at final timepoint 30 hours post treatment. Scale bar = 10 µm. hpt = hours post-treatment. dpt = days post-treatment.

(D) Quantification of length of individual persisting sheaths that showed morphological damage when imaged post-csn treatment, quantified 1 dpt, in Tg(mbp:TRPV1-tagRFPt) animals treated with 1% DMSO, or 5 μM/10 μM csn. Unpaired t-test with Welch's correction. n =11 DMSO-treated cells, n=15 csn-treated cells. Black datapoints represent the mean sheath length of individual cells which were used for statistics, grey datapoints represent individual sheaths. Error bars are mean ± 95% CI.

(E) Quantification of disorder in images of isolated single Mauthner-myelinating oligodendrocytes immediately post-treatment with 1% DMSO or 5 μM csn. Unpaired t-test with Welch's correction, n = 4 DMSO-treated animals, n = 12 csn (5 μM)-treated animals. Error bars are mean ± 95% CI.

(F) Quantification of disorder over time in the same cells in images taken of isolated single Mauthner-myelinating oligodendrocytes immediately post-treatment with 1% DMSO or 5 μM csn and again 1 dpt. n = 4 DMSO-treated animals, n = 12 csn (5 μM)-treated animals.

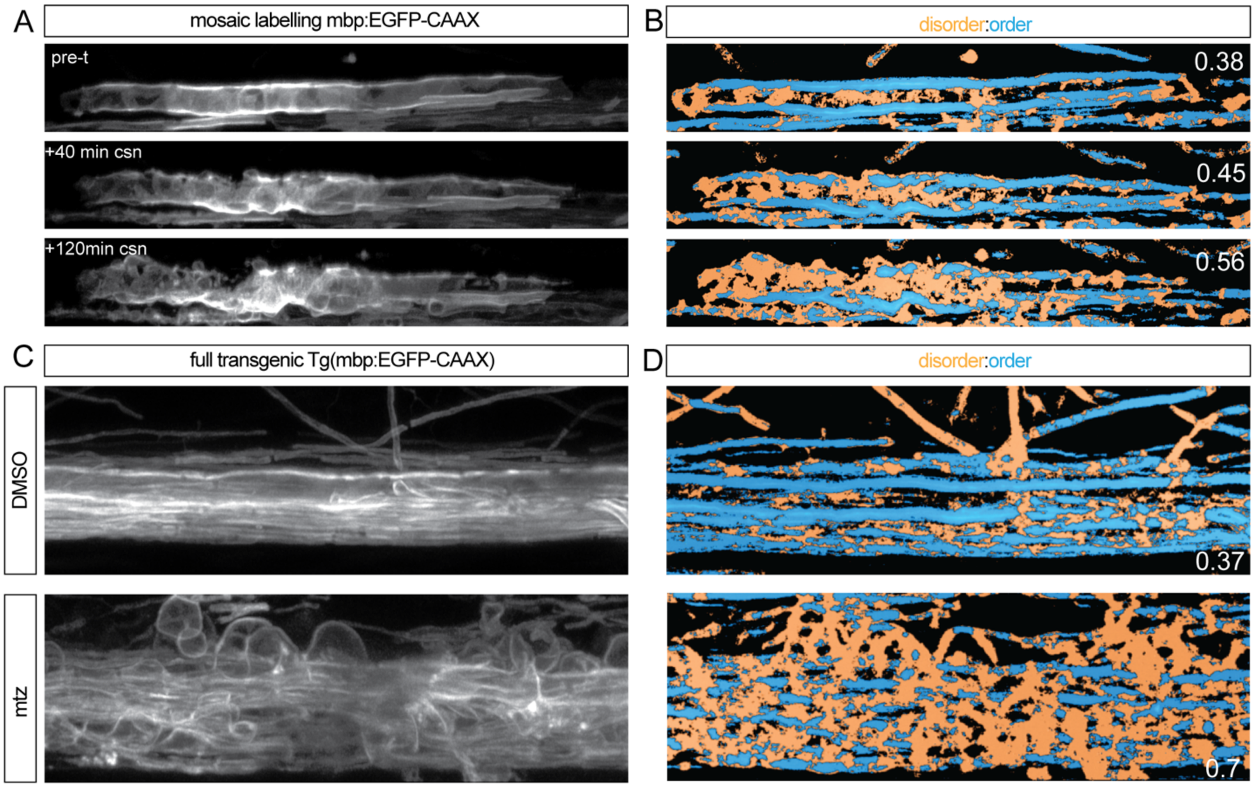

Fig. S5. Automated analysis of myelin damage ('disorder')

(A) Timelapse series of confocal images of a mosaically-labelled oligodendrocyte (mbp:EGFP-CAAX) and its myelin in a Tg(mbp:TRPV1-tagRFPt) animal pre-treatment (pre-t, 4 dpf) with 10 µM csn, 40 minutes into treatment and 120 minutes into treatment.

(B) Machine learning-based detection of ordered myelin (more horizontally aligned pixels, blue) and disordered myelin (less horizontally aligned pixels, orange) from images in (c). Values (referred to as 'disorder' in the text) are a ratio between number of disordered pixels: number of ordered+disordered pixels.

(C) Confocal images of myelin in the ventral spinal cord in a Tg(mbp:EGFP-CAAX; mbp:mCherry-NTR) animal immediately post 2 days of treatment with 1% DMSO or 5 mM mtz.

(D) Machine learning-based detection of ordered myelin (more horizontally aligned pixels, blue) and disordered myelin (less horizontally aligned pixels, orange) from images in (a). Values (referred to as 'disorder' in the text) are a ratio between number of disordered pixels: number of ordered+disordered pixels.

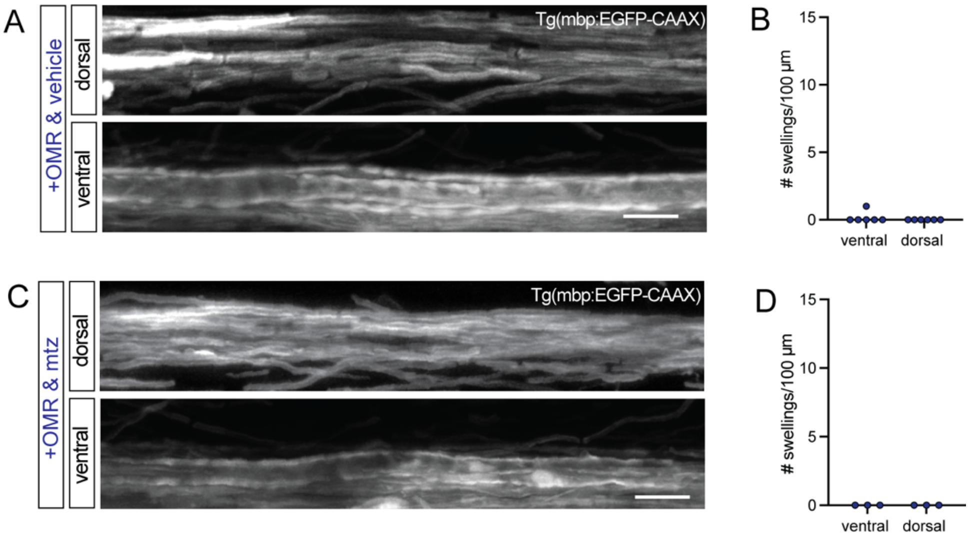

Fig. S6. Stimulation of swimming using the optomotor response for 24 hours does not induce myelin swelling

(A) Confocal images of myelin in the dorsal and ventral spinal cord in a Tg(mbp:EGFP-CAAX; mbp:mCherry-NTR) animal post-24-hour stimulation of swimming using the OMR. Scale bars = 10 µm.

(B) Quantification of the number of swellings in vehicle treated Tg(mbp:EGFP-CAAX; mbp:mCherry-NTR), counted in the dorsal and ventral spinal cord post-24-hour stimulation of swimming using the OMR.

(C) Confocal images of myelin in the dorsal and ventral spinal cord in a Tg(mbp:EGFP-CAAX) animal post-24-hour stimulation of swimming using the OMR and treatment with mtz. Scale bars = 10 µm.

(D) Quantification of the number of swellings in mtz treated Tg(mbp:EGFP-CAAX) animals, counted in the dorsal and ventral spinal cord.

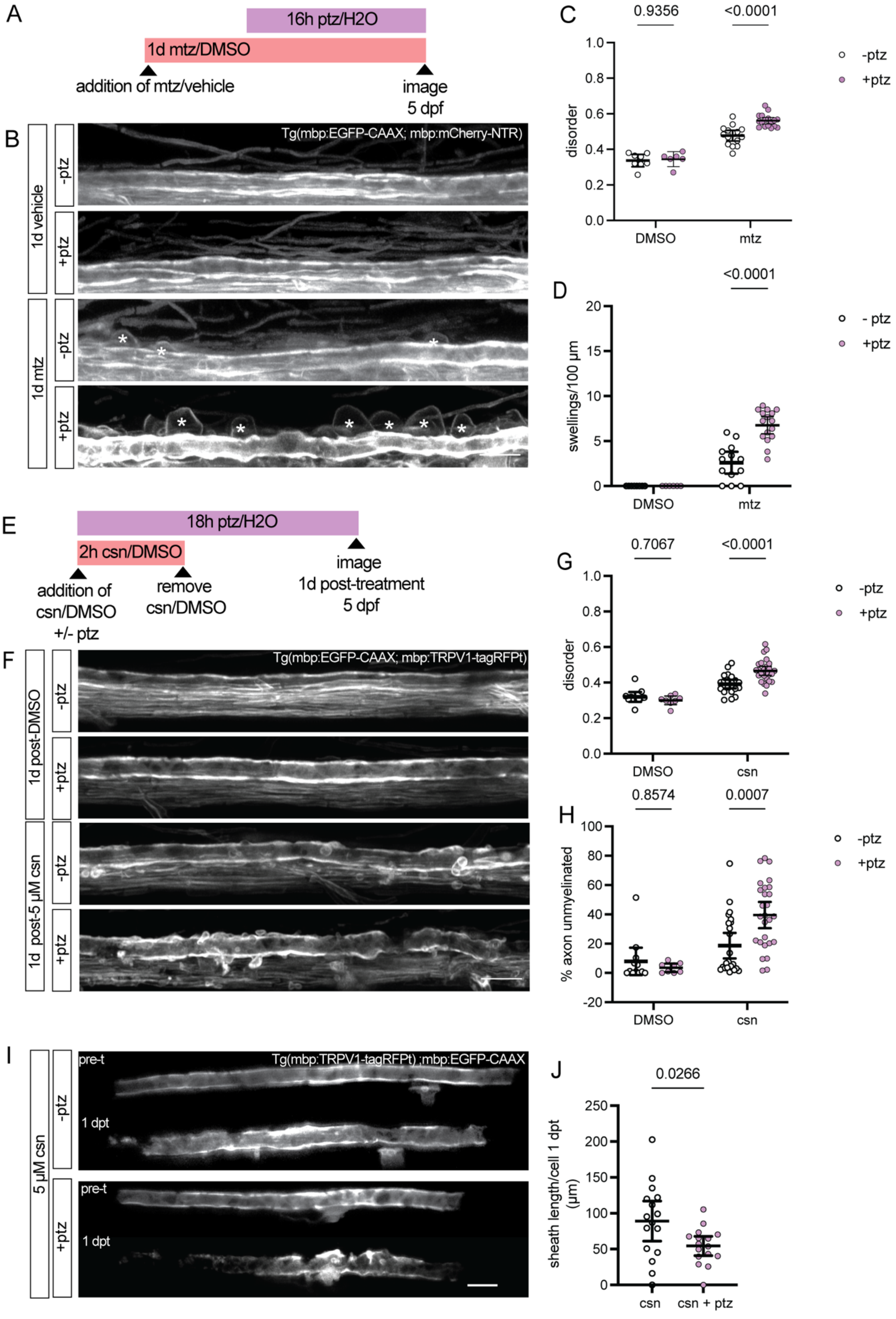
A
16h ptz/H2O
1d mtz/DMSO
addition of mtz/vehicle
image
5 dpf
B
Tg(mbp:EGFP-CAAX; mbp:mCherry-NTR)
1d vehicle
1d mtz
-ptz
+ptz
C
0.9356
<0.0001
disorder
DMSO
mtz
-ptz
+ptz
D
<0.0001
swellings/100 µm
DMSO
mtz
- ptz
+ptz
E
18h ptz/H2O
2h csn/DMSO
addition of csn/DMSO +/- ptz
remove csn/DMSO
image
1d post-treatment
5 dpf
F
Tg(mbp:EGFP-CAAX; mbp:TRPV1-tagRFPt)
1d post-DMSO
1d post-5 µM csn
G
0.7067
<0.0001
disorder
DMSO
csn
H
0.8574
0.0007
% axon unmyelinated
DMSO
csn
I
5 µM csn
pre-t
1 dpt
Tg(mbp:TRPV1-tagRFPt) ;mbp:EGFP-CAAX
J
0.0266
sheath length/cell 1 dpt (µm)
csn
csn + ptz

Fig. S7. Ptz exacerbates myelin swelling/damage in the Tg(mbp:mCherry-NTR) and Tg(mbp:TRPV1-tagRFPt) models

(A) Schematic of experimental set-up for ptz treatment in the Tg(mbp:mCherry-NTR) model.

(B) Confocal images of myelin in the ventral spinal cord in Tg(mbp:EGFP-CAAX; mbp:mCherry-NTR) animals following 1 day of treatment with vehicle (1%DMSO) or 5 mM mtz, +/- treatment with ptz. Asterisks = myelin swellings. Scale bar = 10 µm.

(C) Quantification of disorder in images in full transgenic reporter in (E). Two-way ANOVA with Šídák's multiple comparisons test, n = 8 DMSO-treated animals, n = 6 DMSO + ptz-treated animals, n = 15 mtz-treated animals, n = 17 mtz+ptz-treated animals. Error bars are mean ± 95% CI.

(D) Quantification of the number of swellings visible above the ventral spinal cord in images in full transgenic reporter in (E). Unpaired t-test with Welch's correction (as values for DMSO-treated groups all = 0). Error bars are mean ± 95% CI.

(E) Schematic of experimental set-up for ptz treatment in the Tg(mbp:TRPV1-tagRFPt) model.

(F) Confocal images of myelin in the ventral spinal cord in Tg(mbp:EGFP-CAAX; mbp:TRPV1-tagRFPt) animals 1 day post treatment with 1% DMSO or 5 µM csn, +/- treatment with ptz. Scale bar = 10 µm.

(G) Quantification of disorder in images in full transgenic reporter in (b). Two-way ANOVA with Šídák's multiple comparisons test. n = 11 DMSO-treated animals, n = 8 DMSO + ptz-treated animals, n = 21 csn-treated animals, n = 26 csn+ptz-treated animals. Error bars are mean ± 95% CI.

(H) Quantification of the % of the Mauthner axon un/de-myelinated using the transgenic reporter Tg(mbp:EGFP-CAAX) in Tg(mbp:TRPV1-tagRFPt) animals. Two-way ANOVA with Šídák's multiple comparisons test. n = 11 DMSO-treated animals, n = 8 DMSO + ptz-treated animals, n = 21 csn-treated animals, n = 26 csn+ptz-treated animals. Error bars are mean ± 95% CI.

(I) Confocal images of mosaically-labelled Mauthner-myelinating oligodendrocytes and their myelin from animals treated for 2 hours with 1% DMSO or 5 µM csn, imaged over time pre-treatment and again 1day post-treatment (dpt). Scale bar = 10 µm.

(J) Quantification of Mauthner-myelinating oligodendrocyte sheath length in Tg(mbp:TRPV1-tagRFPt) animals treated with 5 µM csn or 5 µM csn + ptz, 1 dpt. n =

16 csn-treated animals, n = 16 csn + ptz-treated animals. Unpaired T-test with Welch's Correction. Error bars are mean ± 95% CI.

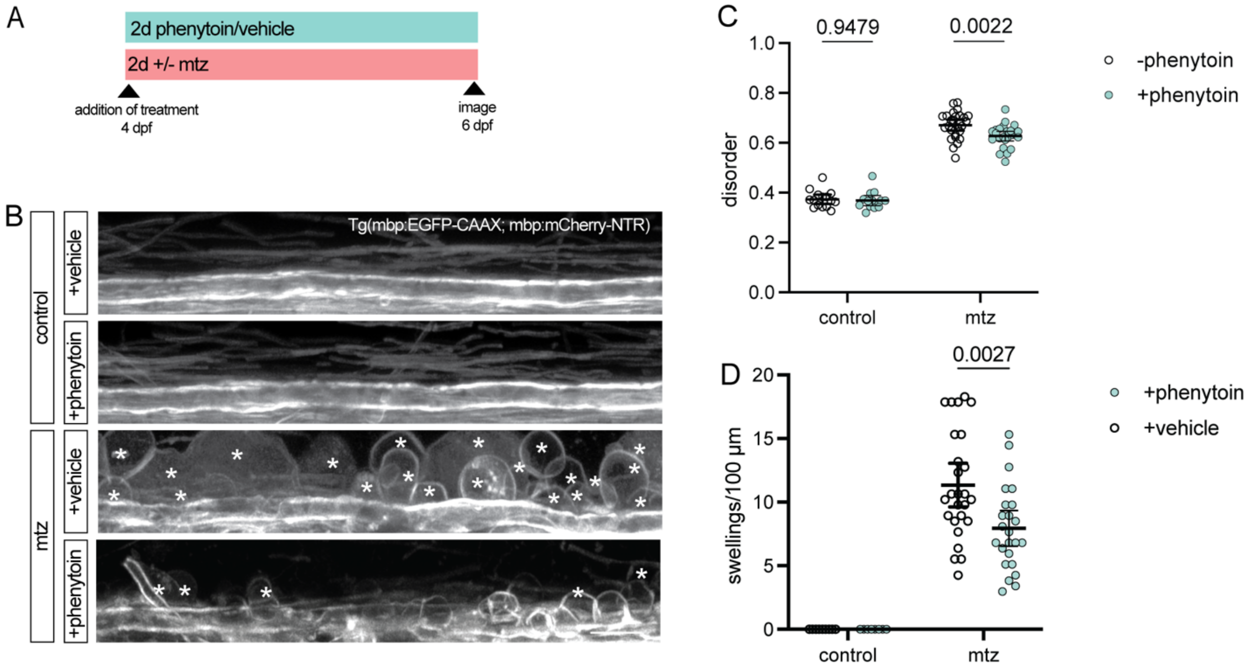

Fig. S8. Phenytoin mitigates myelin swelling in the Tg(mbp:mCherry-NTR) model

(A) Schematic of experimental set-up for phenytoin treatment in the Tg(mbp:mCherry-NTR) model.

(B) Confocal images of all myelin sheaths in the ventral spinal cord labelled by the transgenic reporter Tg(mbp:EGFP-CAAX) from Tg(mbp:mCherry-NTR)-expressing animals treated as controls (with 1% DMSO) or 5 mM mtz for 2 days, with treatment of phenytoin or vehicle for 2 days. Asterisks = swellings. Scale bar = 10 µm.

(C) Quantification of the amount of disordered myelin in the ventral spinal cord post-treatment in controls (n = 15 animals), controls + phenytoin (n = 14 animals), 5 mM mtz only (n = 26 animals), or 5 mM mtz + phenytoin (n = 24 animals). Two-Way ANOVA with Šídák's multiple comparisons test. Error bars are mean ± 95% CI.

(D) Quantification of the number of of swellings in the ventral spinal cord post-treatment with controls only (n =9 animals), controls + phenytoin (n =8 animals), 5 mM mtz only (n = 26 animals), or 5 mM mtz + phenytoin (n = 24 animals). Unpaired t-test with Welch's correction (as values for DMSO-treated groups all = 0).

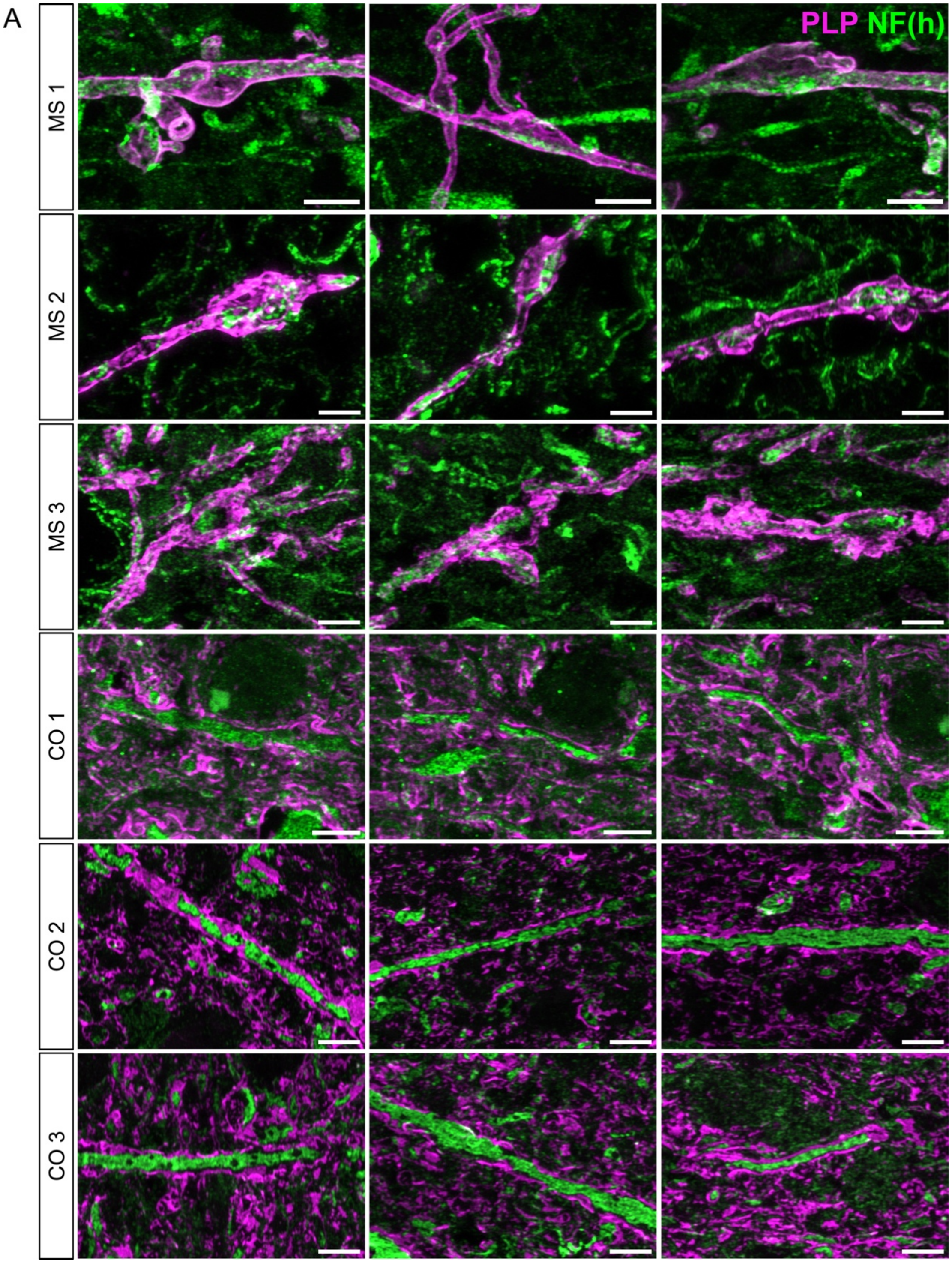

Fig. S9. Additional examples of myelin swelling in MS brain tissue compared to control tissue

(A) Confocal images of myelinated axons examples of perilesional myelin swellings in MS brain tissue (upper - MS) and control white matter (lower - CO). Scale bars = 5 µm.

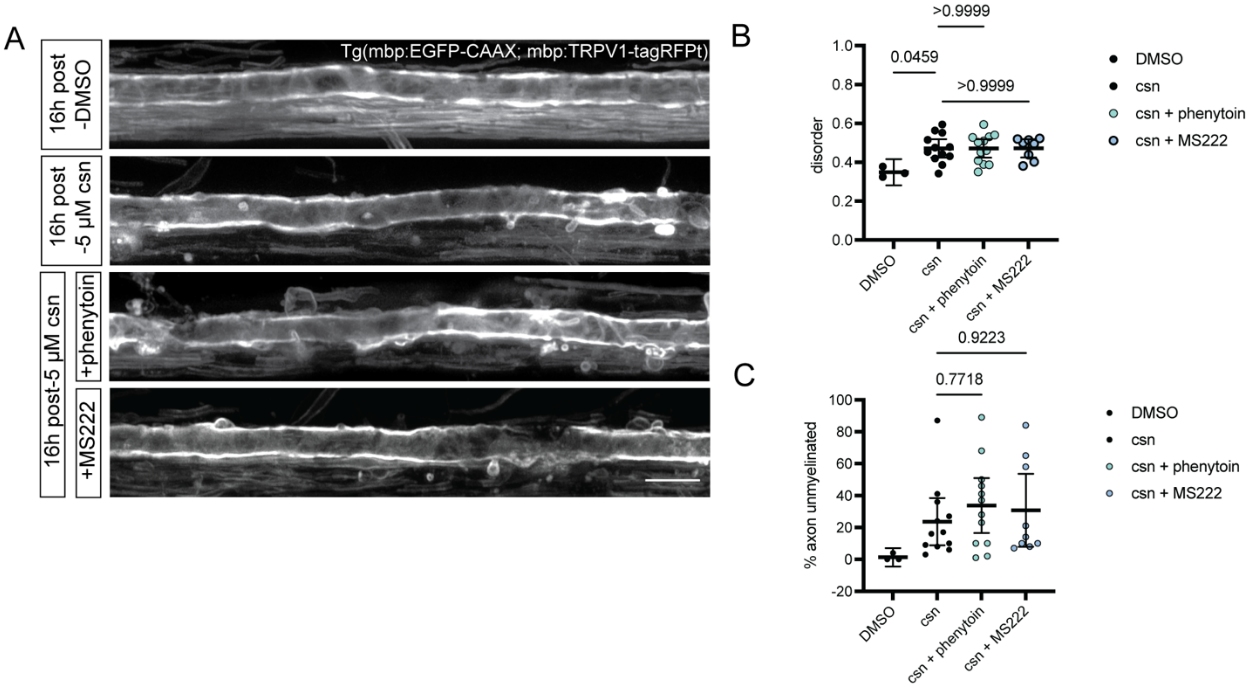

Fig. S10. MS222 and Phenytoin do not affect myelin damage in the Tg(mbp:TRPV1-tagRFPt) model

(A) Confocal images of myelin in the ventral spinal cord in Tg(mbp:EGFP-CAAX; mbp:TRPV1-tagRFPt) animals 1 day post treatment with 1% DMSO or 5 µM csn, +/- treatment with phenytoin or MS222. Scale bar = 10 µm.

(B) Quantification of disorder in images in full transgenic reporter in (a). One-way ANOVA with Tukey's multiple comparisons test, n = 3 DMSO-treated animals, n = 12 csn-treated animals, n = 12 csn+phenytoin-treated animals, n = 8 csn+MS222-treated animals. Error bars are mean ± 95% CI.

(C) Quantification of the % of the Mauthner axon un/de-myelinated using the transgenic reporter Tg(mbp:EGFP-CAAX). One-way ANOVA with Tukey's multiple comparisons test. n = 3 DMSO-treated animals, n = 12 csn-treated animals, n = 12 csn+phenytoin-treated animals, n = 9 csn+MS222-treated animals. Error bars are mean ± 95% CI.

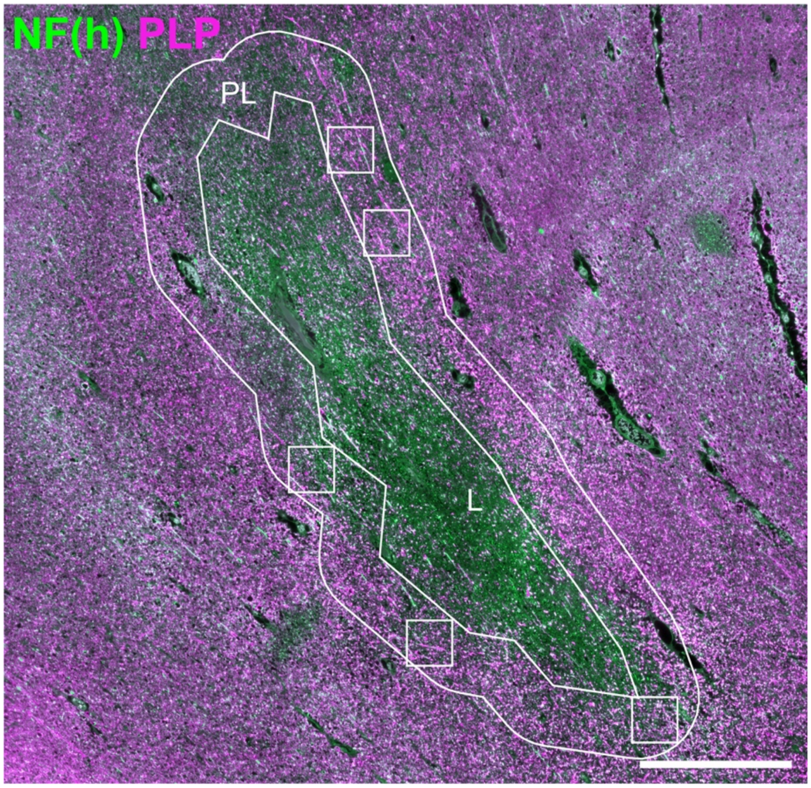

Fig. S11. Examples of areas used for human post-mortem tissue quantification

For quantification of number of swellings in human post-mortem brain tissue, a 200 µm border was made surrounding lesions (or in normally myelinated regions by PLP staining in controls/NAWM), and swellings counted within five 22,500 µm^2 regions (boxes). PL = perilesion. L = lesion (example = chronic active lesion). Scale bar = 500 µm.

ID	Sex	Age (years)	Post-mortem interval (hours)	Diagnosis
CO 1	F	61	NK	NA
CO 2	M	68	10	NA
CO 3	M	86	38	NA
CO 4	F	67	32	NA
CO 5	M	66	NK	NA
CO 6	M	77	22	NA
MS 1	F	57	12	SPMS
MS 2	M	72	11	SPMS
MS 3	F	60	38	SPMS
MS 4	F	57	14	SPMS
MS 5	M	53	14	SPMS
MS 6	F	59	8	SPMS
MS 7	M	50	24	SPMS

Table S1. Human case information from Edinburgh Brain Bank

ID	Sex	Age (years)	Post-mortem interval (hours)	Diagnosis
MS8	F	75	6	PMS
MS9	M	58	8	PMS
MS10	F	44	6	PMS

Table S2. Human case information from Netherlands Brain Bank

Movie S1. Z-stack through zebrafish spinal cord showing that myelin sheath swelling does not surround neuronal cell bodies. Confocal z-stack series showing myelin by Tg(mbp:EGFP-CAAX) green and neuronal cell bodies by Tg(NBT:DsRed) in magenta in the Tg(mbp:mCherry-NTR) model following 2 days of treatment with mtz.

Movie S2. The optomotor response induces swimming in zebrafish. Zebrafish swimming in response to moving grids projected below the petri dish that elicit the optomotor response.

Movie S3. Myelin swelling is dynamic in a mammalian model of demyelination. Live 2-photon time-lapse imaging of myelin in a mouse cortical organotypic slice culture model, illustrating examples of myelin swelling formation and reduction post LPC application. Yellow asterisks point to swelling formation, orange asterisks point to swelling reduction

Movie S4. Myelin swelling is dynamic in postmortem MS tissue. Third harmonic imaging timelapse in postmortem MS tissue illustrating a myelin swelling reducing in size over time.

Movie S5. Myelin swellings in perilesional MS brain tissue. Overview of tile-scanned MS-tissue section zooming in to reveal examples of myelin swellings along axons surrounding a white matter lesion. Immunofluorescence for PLP/myelin (magenta) and neurofilament/axons (green).

Data S1. Separate file: Data-and-analysis-File.xlsx

File documenting data associated with all figures and supplementary materials of the manuscript and corresponding analyses.

Data S2. Separate file: Reproducibility checklist
